# AI in Cytopathology: A Narrative Umbrella Review on Innovations, Challenges, and Future Directions

**DOI:** 10.3390/jcm13226745

**Published:** 2024-11-09

**Authors:** Daniele Giansanti

**Affiliations:** Centro TISP, Istituto Superiore di Sanità, Via Regina Elena 299, 00161 Rome, Italy; daniele.giansanti@iss.it

**Keywords:** cytopathology, artificial intelligence, AI, histopathology, radiology

## Abstract

The integration of artificial intelligence (AI) in cytopathology is an emerging field with transformative potential, aiming to enhance diagnostic precision and operational efficiency. This umbrella review seeks to identify prevailing themes, opportunities, challenges, and recommendations related to AI in cytopathology. Utilizing a standardized checklist and quality control procedures, this review examines recent advancements and future implications of AI technologies in this domain. Twenty-one review studies were selected through a systematic process. AI has demonstrated promise in automating and refining diagnostic processes, potentially reducing errors and improving patient outcomes. However, several critical challenges need to be addressed to realize the benefits of AI fully. This review underscores the necessity for rigorous validation, ongoing empirical data on diagnostic accuracy, standardized protocols, and effective integration with existing clinical workflows. Ethical issues, including data privacy and algorithmic bias, must be managed to ensure responsible AI applications. Additionally, high costs and substantial training requirements present barriers to widespread AI adoption. Future directions highlight the importance of applying successful integration strategies from histopathology and radiology to cytopathology. Continuous research is needed to improve model interpretability, validation, and standardization. Developing effective strategies for incorporating AI into clinical practice and establishing comprehensive ethical and regulatory frameworks will be crucial for overcoming these challenges. In conclusion, while AI holds significant promise for advancing cytopathology, its full potential can only be achieved by addressing challenges related to validation, cost, and ethics. This review provides an overview of current advancements, identifies ongoing challenges, and offers a roadmap for the successful integration of AI into diagnostic cytopathology, informed by insights from related fields.

## 1. Introduction

### 1.1. The Impact of Digitalization Cytopathology: Transforming Diagnostic Practices

Cytopathology is the branch of medicine and biology focused on studying cells to diagnose diseases, especially by analyzing cellular changes that could indicate conditions such as cancer. This involves using microscopes to examine cell samples from smears, aspirations, or biopsies to identify diseases and support clinical diagnoses [1,2,3].

In recent years, digital health technologies have shown the potential to transform cytopathology, with some improvements in the accuracy and efficiency of analyses. Digital imaging, for instance, enables the scanning of slides at high resolution, which can lead to more precise examinations and better diagnostic accuracy. However, the full extent of this improvement and its widespread impact remain subjects of ongoing study [4].

Digital tools also offer the possibility of remote consultations and second opinions, promoting global collaboration among specialists. While these advances could streamline workflows and boost productivity, their overall efficacy in supporting personalized patient care through detailed cytopathological data are still being evaluated. As of now, the integration of digital health in cytopathology presents promising, yet still evolving, benefits [5,6].

Key potential benefits include [5,6]:

Enhanced Diagnostic Accuracy: High-resolution imaging may improve the detection of subtle cellular abnormalities, though the level of precision in all cases is still under review [7].

Remote Consultations and Telepathology: Pathologists can conduct remote consultations and second opinions, though the reliability and accessibility of such methods vary [7,8].

Streamlined Workflow: While automating tasks such as slide management and data entry has the potential to enhance lab efficiency, its widespread adoption and real-world impact are still uncertain [9].

Long-term Data Storage: Digital storage of slides allows for secure, long-term management, but concerns about data integrity and retrieval over extended periods remain.

Integration with EHRs: The integration with electronic health records could improve care coordination, though seamless functionality across different systems remains a challenge.

Educational Tools: Virtual slides and interactive modules support training, yet their effectiveness compared with traditional methods is still being assessed.

Quality Assurance and Standardization: While standardized protocols and quality control measures could enhance consistency, there is ongoing discussion about the implementation of such measures across different laboratories.

Research and Data Analysis: Digital tools can help aggregate and analyze large datasets, but their utility in advancing research on disease patterns and new diagnostic methods requires further evidence.

The evolving nature of these technologies highlights both their potential and the need for continued evaluation.

The potential of digitalization and AI in cytopathology is significant, but several challenges need to be addressed to realize these advancements fully [10,11]. Cytopathology, dissimilar to radiology, has experienced a slower integration with the digital health landscape, which can be attributed to several factors [12,13]. One of the key issues is the complexity of cytopathological samples. Cytopathology requires the detailed and often intricate evaluation of individual cells, making it more challenging to automate compared with radiological images, which are generally more uniform and structural. For example, replicating the focus function in cytopathology is highly complex and demands substantial memory resources. Another contributing factor is the difference in imaging technologies. Radiology has benefited from rapid technological advancements, particularly with systems such as CT and MRI, which have facilitated swift digitalization and integration into clinical workflows, largely thanks to the rapid adoption of the DICOM standard [14]. In contrast, the development of a DICOM standard for cytopathology (DICOM WSI) has been slower and more complex [15,16]. Additionally, the diagnostic process in cytopathology involves finer and more interpretative work compared with radiology. The digitalization of cytopathology, therefore, faces specific technical and clinical challenges, including the need to interpret intricate cellular details and manage the variability of cell samples. Standardization presents yet another challenge. Radiology has benefited from well-established standards and protocols, while cytopathology remains more diversified and less standardized, which has contributed to the slower adoption of digital technologies in this field. However, despite these obstacles, cytopathology is progressively integrating digital technologies and advanced tools, and it is anticipated that this evolution will continue in the coming years.

### 1.2. Integrating Artificial Intelligence in Digital Cytopathology

Artificial intelligence (AI) is revolutionizing many areas of medicine, and cytopathology is no exception. Traditionally, cytopathology, which focuses on diagnosing diseases through cellular samples, has relied heavily on the expertise of pathologists examining slides under a microscope. However, the integration of AI into cytopathology is poised to bring significant advancements, aligning with broader objectives in digital pathology, such as enhancing diagnostic accuracy, improving workflow efficiency, and advancing patient care [17,18]. The insights provided in studies referenced by [17,18] underscore the transformative potential of AI technologies in cytopathology.

AI technologies, especially those based on machine learning (ML) and deep learning (DL), are increasingly being adopted in cytopathology. These advanced algorithms are capable of processing large volumes of cytological data swiftly and with high precision. By leveraging extensive datasets of annotated images, AI can assist in identifying cellular abnormalities, classifying cell types, and even predicting disease progression. This capability has the potential to complement and enhance the diagnostic process, allowing pathologists to detect subtle patterns and anomalies that may be missed in traditional evaluations.

According to the studies cited in [17,18], the adoption of AI in cytopathology offers several transformative benefits. First, AI tools can significantly improve diagnostic accuracy by providing consistent and objective analyses, thereby reducing variability and human error. Additionally, these tools can streamline diagnostic workflows by automating routine tasks such as image sorting and preliminary screening, which helps expedite the diagnostic process and alleviates the workload on cytopathologists. Moreover, AI supports the training and education of new cytopathologists by offering interactive learning modules and access to a broad range of case studies.

Furthermore, AI integration enhances access to cytopathology services by enabling remote consultations and fostering collaboration through digital platforms. This capability is particularly valuable in underserved areas or for institutions seeking expert second opinions from specialists located in other regions.

However, as noted in [17,18,19], the integration of AI into cytopathology also presents several challenges. Effective implementation requires seamless integration with existing diagnostic workflows and the establishment of standardized protocols to ensure compatibility across different systems. Additionally, important regulatory and ethical considerations need to be addressed, such as the rigorous validation of AI tools, concerns regarding data privacy, and the risk of over-reliance on machine-assisted diagnoses. Furthermore, there may be resistance from practitioners accustomed to traditional methods, who will need time to adapt to these new technologies.

### 1.3. Purpose of the Study

The integration of AI into the healthcare domain holds transformative potential with profound implications for cytopathology. AI has the capacity to revolutionize diagnostic processes, enhance accuracy, and streamline workflows while also presenting strategic challenges that must be addressed. An overview of reviews can provide a holistic view of how AI is reshaping cytopathology, revealing both the advancements and obstacles encountered in this integration.

The purpose of this study is to develop a narrative umbrella review to comprehensively explore the role of artificial intelligence (AI) in cytopathology. This review seeks to aggregate and synthesize findings from existing reviews to offer a consolidated understanding of how AI is being applied within the field. By identifying and analyzing the key themes and trends emerging from these studies, we aim to highlight significant advancements, such as improvements in diagnostic precision and workflow efficiency, as well as the challenges that have arisen, including issues related to implementation, standardization, and ethical considerations.

Specific aims:

1. Themes and categorization: Identify and organize the main themes emerging from existing studies, with a focus on how AI is being utilized to enhance cytopathological diagnosis.
2. Opportunities and challenges: Analyze the opportunities and challenges encountered in the field, examining benefits related to diagnostic precision and workflow optimization alongside the difficulties faced during implementation.
3. Emerging recommendations: Provide recommendations based on this review’s findings, with a particular focus on critical issues such as practical implementation, standardization of procedures, and ethical aspects related to the use of AI in cytopathology.

## 2. Methods

An umbrella review (narrative review of reviews) was conducted, focusing on the field of the intersection of cytopathology with AI. A standardized checklist for narrative reviews, the ANDJ Narrative Checklist available online [20] was used. The search was based on targeted searches on Google Scholar, PubMed, and Scopus. 

### 2.1. Search Strategies

The search was performed both in the [Title/abstract] and in the full text.

The components of this overview were obtained by means of the combination of two groups of keywords also combined with AND/OR Boolean logic of a search:

- Machine Learning in Cytopathology
- Deep Learning Algorithms
- Digital Imaging in Cytopathology
- Automated Diagnostic Systems
- Cell Image Analysis
- AI-assisted Diagnosis
- Computer-Aided Diagnosis
- Predictive Analytics in Cytopathology
- Artificial Intelligence in Cellular Diagnostics
- Digital Pathology Tools
- Algorithmic Classification of Cells
- AI-based Cytological Screening
- Machine Learning for Cellular Abnormalities
- Image Recognition in Cytopathology
- AI-driven Cytopathological Innovation
- Diagnostic Precision with AI
- AI in Medical Imaging
- Remote Cytopathology Consultations
- Cytopathological Workflow Optimization
- AI and Pathologist Collaboration

Table 1 shows the research areas related to the application of artificial intelligence (AI) and machine learning (ML) in cytopathology, including key themes and their corresponding focused research queries. Each theme highlights the potential of AI in improving the accuracy and efficiency of cytopathological diagnostics, ranging from deep learning algorithms for cell analysis to the optimization of workflow in cytopathology.

### 2.2. Assessment Criteria for Study Inclusion

To ensure a rigorous and high-quality narrative review, each selected study was assessed based on the following criteria:

Clarity of Rationale (N1): This criterion evaluates whether the study clearly articulates the reason for its investigation. The rationale should define the research problem, highlight its significance, and explain why the study is necessary. A well-defined rationale provides context and justifies the research effort. 

Design Appropriateness (N2): This criterion assesses whether the study’s design is suitable for answering the research question or hypothesis. The design should align with the objectives and scope of the study. Appropriate design includes selecting the right methodology, sample size, and data collection methods.

Methodological Clarity (N3): Methodological clarity refers to the extent to which the study’s methods are described in detail and are replicable. This includes the transparency of procedures for data collection, analysis, and interpretation. The study should provide clear information on how data were gathered, the tools and techniques used, and how the analysis was conducted. This clarity ensures that the study can be reproduced or critiqued based on the methodology described.

Result Presentation (N4): This criterion evaluates how effectively the study presents its findings. Results should be clearly organized, accurately reported, and appropriately interpreted. The presentation should include relevant tables, figures, and statistical analyses that support the conclusions drawn. The clarity of the result presentation allows readers to understand and evaluate the study’s outcomes.

Justification of Conclusions (N5): This criterion assesses whether the study’s conclusions are supported by its results. The study should provide a logical link between presented data and the conclusions drawn. It should discuss the implications of the findings, address limitations, and suggest areas for future research. The justification of conclusions ensures that the study’s outcomes are valid and that the conclusions are based on sound evidence.

Disclosure of Conflicts of Interest (N6): Disclosure of conflicts of interest is crucial for assessing the impartiality and credibility of the study. This criterion checks whether the authors have declared any financial, professional, or personal interests that could bias the research. Full disclosure helps in evaluating the objectivity of the study and ensures that the findings are not influenced by external pressures or biases.

The choice of the component elements of this overview was made taking into account the 5 parameters (N1–N5) evaluated with a score from 1 = minimum to 5 = maximum and one parameter (N6) with a binary assessment (Yes/No). These parameters have been identified into the following:

All the selected studies had to have the parameter N6 with “Yes” and the parameters N1-N5 with a score >3.

In addition to these parameters, in this umbrella review, we have also given weight to the publication date, prioritizing more recent reviews if the topic has been covered in later publications. In other words, we have established a sort of time-based priority to ensure that the most current and relevant findings are considered. This approach helps us capture the latest advancements and developments in the field while also maintaining a comprehensive overview of the existing literature.

**Figure 1:**
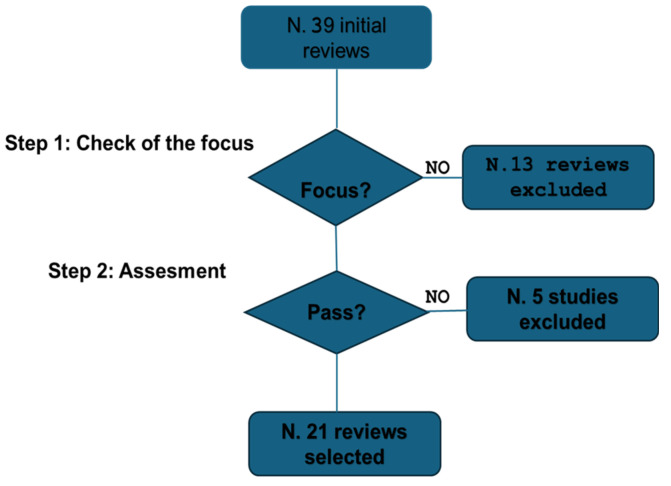
Details on the process of study selection.

The procedure ultimately identified 21 studies at the end of the selection process. Figure 1 outlines all the steps involved. This figure illustrates that the initial search yielded a total of 39 studies. From these, 13 studies were excluded because of their poor focus on AI. Following the evaluation according to the methodology described above, 21 studies were retained for further consideration, while 5 studies were excluded. See Section S1 for the narrative checklist.

## 3. Results

In total, 21 studies were selected [21,22,23,24,25,26,27,28,29,30,31,32,33,34,35,36,37,38,39,40,41], comprising all the relevant research identified on PubMed.

The results are systematically organized into multiple subsections to provide a comprehensive understanding of the findings.

Section 3.1 reports a synoptic diagram of the results.

Section 3.2 presents trends observed across the studies, enhanced by graphical representations that illustrate these data visually. This subsection aims to highlight overarching patterns and significant shifts within the field, offering a clear view of how the research landscape has evolved over time.

Section 3.3 aligns the outcomes with the specific objectives and is therefore divided into three subsections, each corresponding to a specific sub-aim.

Section 3.3.1 answers sub-aim (1: *“Themes and categorization: Identify and organize the main themes emerging from existing studies, with a focus on how AI is being utilized to enhance cytopathological diagnosis”*). This subsection explores:

(a) Extraction of common themes and interconnections among the studies, highlighting recurring messages and drawing correlations between research findings. This allows for a synthesis of insights that reflect broader implications within the literature.
(b) A detailed categorization of the studies, distinguishing fine-grained thematic areas as well as broader categories. This categorization organizes research into specific subfields and general domains, offering a comprehensive view of the diverse aspects covered by the studies and highlighting key areas of focus and specialization.

Section 3.3.2 answers sub-aim (2: “*Opportunities and challenges: Analyze the opportunities and challenges encountered in the field, examining benefits related to diagnostic precision and workflow optimization alongside the difficulties faced during implementation*”). This subsection discusses the potential for AI to enhance diagnostic precision and improve workflow efficiency while addressing challenges such as data variability, technical integration issues, and the need for resource investment during the implementation phase.

Section 3.3.3 addresses sub-aim (3: “*Emerging recommendations: Provide recommendations based on the review findings, with particular focus on critical issues such as practical implementation, standardization of procedures, and ethical aspects related to the use of AI in cytopathology*”). It synthesizes key recommendations from this review, emphasizing the need for standardized procedures to facilitate AI integration into clinical workflows. Additionally, it highlights practical challenges in implementation, such as data management and resource allocation, and underscores the importance of addressing ethical issues such as transparency, data privacy, and algorithmic biases in AI-driven cytopathology.

Analytical summaries of the overviewed reviews [21,22,23,24,25,26,27,28,29,30,31,32,33,34,35,36,37,38,39,40,41] are also reported in the Supplementary Materials.

### 3.1. Synoptic Diagram

**Figure 2:**
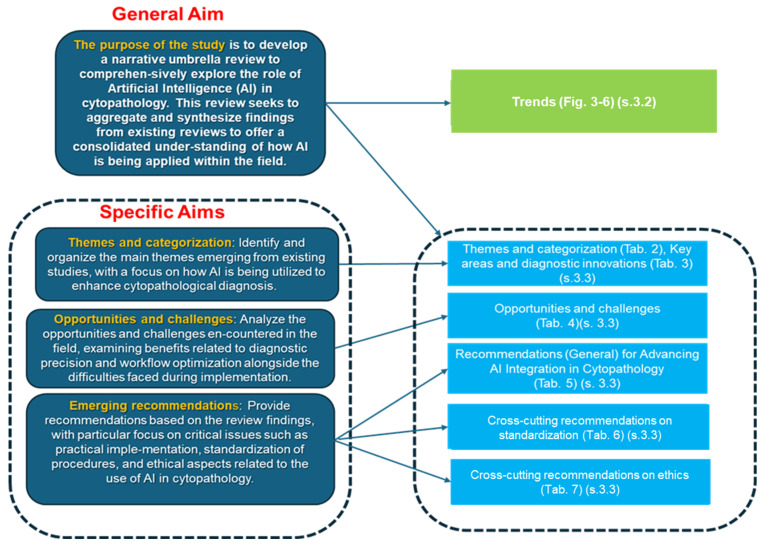
Synoptics diagram reporting a sketch of the the results.

The diagram in Figure 2 provides a highly concise sketch of the results organized into tabular connections and diagrams aligned with the overall aim and specific objectives.

**Figure 3:**
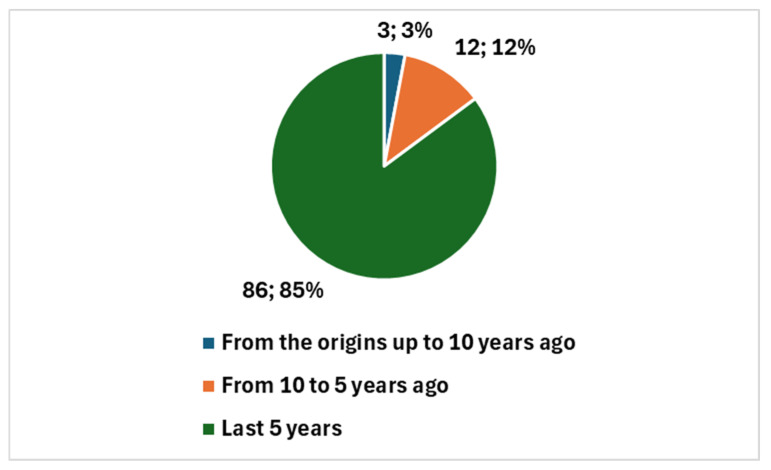
Temporal trend of studies focusing on the integration of AI with digital cytopathology.

**Figure 4:**
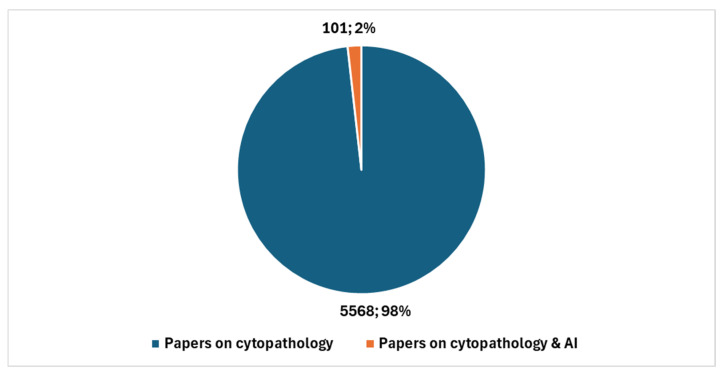
Studies in cytopathology that do not involve AI compared with studies in cytopathology focused on AI.

**Figure 5:**
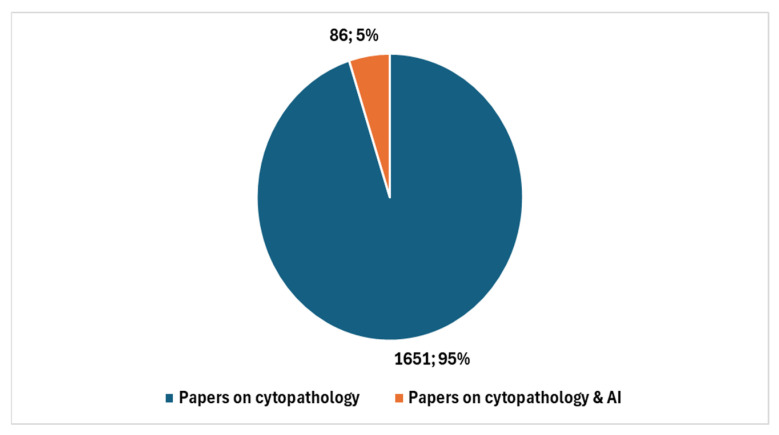
Studies in cytopathology that do not involve AI compared with studies in cytopathology focused on AI with reference to the last five years.

**Figure 6:**
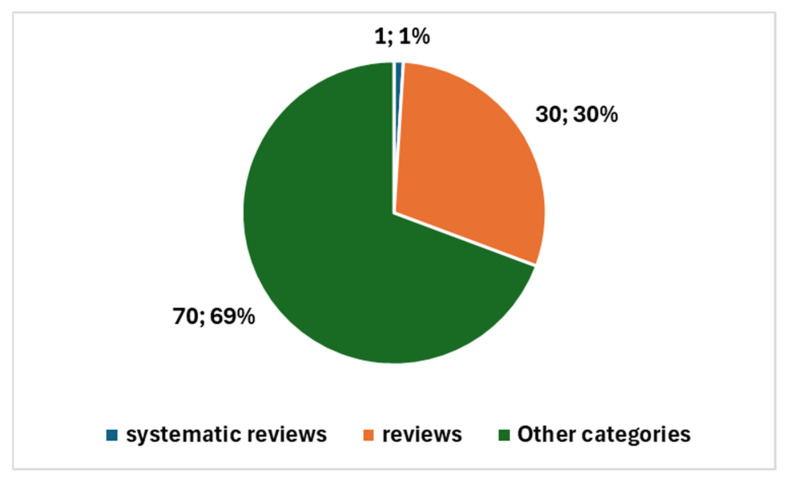
Proportion of studies in cytopathology and AI.

Block 1 (from top to bottom) highlights trends supported by the diagrams in Figure 3, Figure 4, Figure 5 and Figure 6. 

Block 2 references both Table 2, which emphasizes the themes and the categorization emerging in the fields, and Table 3 reporting the main areas with the emerging diagnostic innovations. Block 3 focuses on the opportunities and challenges, as shown in Table 4.

Block 4 faces the emerging recommendations as presented in Table 5.

Blocks 5 and 6 deepen the analysis facing both the ethical and standardization issues emerging as cross-cutting recommendations form the analysis reported in Table 6 and Table 7, respectively.

### 3.2. The Trends in the Studies on AI in the Field of Cytopathology

A search conducted on PubMed on 15 July 2024 (when the overview started), using the composite keyword listed in position 1 of Box 1, returned 101 articles. It is intriguing to analyze both the temporal evolution of the scientific output and its proportion relative to the overall studies in cytopathology, as well as the types of studies produced.

The 101 articles were published starting in 1998, a period when discussions were more focused on neural networks rather than true artificial intelligence (AI). Notably, 98 of these studies, representing 97.0%, were published in the last decade. Furthermore, 86 of these studies, accounting for 85.1%, were published in the past five years, a period notably influenced by the COVID-19 pandemic.

The impact of the COVID-19 pandemic on research output in this period has been profound. The pandemic has accelerated the pace of research, particularly in fields where rapid technological advancements were crucial. The surge in AI-related studies within cytopathology reflects a broader trend driven by the urgent need for innovative solutions to manage the healthcare crisis. The pandemic has highlighted the critical role of AI in enhancing diagnostic capabilities, managing large volumes of data, and facilitating remote consultations. This heightened focus on digital health solutions has contributed to the significant increase in AI research output during the past five years.

In the broader context of cytopathology research, a total of 5669 studies have been produced (key in position *2*, Box 1). Therefore, studies specifically addressing AI represent 1.79% of the total. Figure 3 reports the temporal trend of studies focusing on the integration of AI with digital cytopathology.

Figure 4 shows the number of studies in cytopathology not involving AI compared with the number of studies in cytopathology focused on AI.

It is noteworthy that a comparison limited to the past five years reveals a significant increase in studies conducted in cytopathology and AI. Specifically, a comparison between the total number of cytopathology studies over the last five years, which amounts to 1737, and those focused on AI, which total 86, results in a ratio of 4.95. Figure 5 illustrates, with reference to the past five years, the number of studies in cytopathology not involving AI compared with the number of studies centered on AI.

The COVID-19 pandemic has notably accelerated the focus on AI within the field of cytopathology. This is evidenced by the increased percentage of studies concentrating on AI, reflecting a surge in research and technological advancements prompted by the pandemic. The need for rapid, efficient diagnostics and remote consultation solutions drove this trend. The integration of AI technologies helped address the unprecedented challenges posed by COVID-19 by improving diagnostic accuracy, managing large volumes of patient data, and facilitating remote medical consultations.

The increased emphasis on AI during this period highlights its critical role in enhancing diagnostic capabilities and adapting to new healthcare demands. This shift not only underscores the growing importance of digital health innovations but also demonstrates how AI has become an essential component in advancing cytopathological practices in response to global health crises.

The types of studies produced are illustrated in Figure 6. This includes one systematic review and 30 reviews of various types, in addition to the remaining 70 primary studies. This distribution underscores the growing interest in AI applications within cytopathology and reflects the diverse nature of the research being conducted, influenced significantly by the pandemic-driven emphasis on technological innovation in healthcare.

Box 1 **The proposed composite keys.**

*(Cytopathology [Title/Abstract]) AND ((Artificial intelligence [Title/Abstract]) OR (machine learning [Title/Abstract]) OR (deep learning [Title/Abstract]) OR (neural network [Title/Abstract]))*

*((Cytopathology [Title/Abstract])*

### 3.3. Outcome from the Umbrella Review Aligned with the Specific Aims

#### 3.3.1. Themes and Categorization

Table 2 reports a categorization with a brief description of each contribution and the focus/role of the AI.

The integration of artificial intelligence (AI) into diagnostic medicine is profoundly transforming the field, as evidenced by recent studies [21,22,23,24,25,26,27,28,29,30,31,32,33,34,35,36,37,38]. The research collectively underscores how AI is enhancing diagnostic practices across various domains.

Studies such as those by Ciaparrone et al. [21] and Kim et al. [25,26] highlight the transformative role of AI in diagnostic automation and digital cytology. Ciaparrone et al. [21] focus on the impact of AI on urine cytology, demonstrating its potential to improve diagnostic speed, cost-efficiency, and accuracy by automating image analysis. Similarly, Kim et al. [25,26] provide comprehensive reviews on AI applications in digital cytology, offering guidelines for integrating AI technologies to enhance workflow and diagnostic precision.

The advancements in cancer diagnosis are notably reflected in the work of Zhang et al. [22] and Malik & Zaheer [27]. Zhang et al. [22] explore the potential of AI in detecting early markers of thyroid cancer, emphasizing its role in improving diagnostic accuracy and early detection. Malik & Zaheer [27] analyze the use of AI-driven tools such as ChatGPT to support cancer diagnosis, showcasing how conversational AI can integrate with traditional diagnostic processes.

The ability of AI to complement traditional diagnostic methods is also highlighted by Giovanella et al. [24] and Wong et al. [32]. Giovanella et al. [24] review how AI enhances the diagnostic accuracy and risk assessment of thyroid nodules when used alongside traditional methods such as ultrasound and fine needle aspiration (FNAC). Wong et al. [32] provide an overview of machine learning advancements in thyroid cytopathology, illustrating the integration of AI into established diagnostic practices. Furthermore, Ludwig et al. [33] update on recent advancements in AI for diagnosing and classifying thyroid nodules, reflecting how AI technologies are being incorporated into thyroid diagnostics.

In addition, Tessler et al. [35] review AI tools for thyroid nodule diagnosis with a particular focus on malignancy.

Specialized applications of AI are explored by Singla et al. [30] and Sunny et al. [31]. Singla et al. [30] focus on the role of AI in fungal cytology, highlighting its potential to improve the detection and classification of fungal infections. Sunny et al. [31] discuss the application of AI in analyzing CD44-SNA1 markers for identifying high-grade dysplastic and neoplastic oral lesions, demonstrating the ability of AI to enhance diagnostic precision in specific contexts.

In the realms of microbiological and pancreatic pathology, Marletta et al. [34,35] and Hameed & Krishnan [36] provide insights into the impact of AI. Marletta et al. [34]. Hameed & Krishnan [36] examine the effectiveness of AI in diagnosing pancreatic cancer, discussing both the benefits and challenges of AI-driven diagnostic tools.

Thakur et al. [37] offer a systematic review of AI applications in non-gynecological cancer cytopathology, while Alrafiah [38] assesses the overall performance and impact of AI technologies in cytopathology. These studies underscore the growing influence of AI in enhancing diagnostic accuracy, efficiency, and integration with traditional methods across various diagnostic fields. Finally, Vaickus et al. [39] review AI advancements in cytopathology and their integration with consensus rule sets, while Jorda et al. [40] discuss the role of AI in enhancing diagnostic accuracy in urinary tract cytopathology and Velez Torres et al. [41] examines the impact of AI on thyroid fine-needle aspiration cytology and risk assessment.

The body of research from [21] to [41] illustrates the transformative potential of AI in modern diagnostic medicine, showcasing its ability to refine diagnostic practices, enhance precision, and integrate with established methods.

**Table 2:** Summary of Recent Studies on AI Applications in Cytopathology: Focus, Impact, and Categorization.

Reference	Brief Description	Focus on AI	Categorization
Ciaparrone et al. (2024) [21]	This study evaluates computer-assisted urine cytology, emphasizing the role of AI in enhancing diagnostic speed, reducing costs, and improving accuracy. It examines how automation and AI-driven image analysis contribute to a more efficient diagnostic process.	Investigates AI in automating image analysis for urine cytology, aiming to speed up the diagnostic process and reduce costs.	AI in Diagnostic Automation
			
Zhang et al. (2024) [22]	Explores the role of AI in diagnosing early markers for thyroid cancer, focusing on how AI algorithms improve diagnostic precision. The paper highlights the potential of AI in identifying early cancer markers crucial for timely and accurate diagnosis.	Focuses on the role of AI in identifying and diagnosing early markers for thyroid cancer, improving accuracy and early detection.	AI in Cancer Diagnosis
			
Caputo et al. (2024) [23]	Discusses how AI and computational advancements are transforming cytopathology, with insights from emerging pathologists. The paper highlights the integration of AI in modern cytopathology practices, contributing to more precise diagnostics.	Highlights the integration of AI and computational methods in modern cytopathology, reflecting on their transformative effects on the field.	AI and Computational Integration
			
Giovanella et al. (2024) [24]	Reviews how AI integrates with traditional diagnostic methods for evaluating thyroid nodules, such as ultrasound and fine needle aspiration (FNAC). The study focuses on the role of AI in enhancing diagnostic accuracy and risk assessment.	Reviews the role of AI in integrating diagnostic tools such as ultrasound and FNAC, aiming to improve overall diagnostic accuracy and patient management.	AI in Integrated Diagnostics
			
Kim et al. (2024) [25]	Part two of a review on digital cytology focuses on AI applications in cytology practices. It offers guidelines and recommendations for incorporating AI technologies to enhance diagnostic workflows.	Examines AI technologies in digital cytology and offers recommendations for their implementation to improve diagnostic practices.	AI in Digital Cytology
			
Kim et al. (2024) [26]	The first part of a review series on digital cytology addresses the practical aspects of implementing AI tools in clinical settings. It discusses various AI tools and their benefits in enhancing cytology practices.	Discusses the implementation of AI tools in digital cytology, including practical considerations and potential benefits for clinical practice.	AI in Digital Cytology
			
Malik & Zaheer (2024) [27])	Analyze how ChatGPT and similar AI-driven tools can support cancer diagnosis. The study evaluates the integration of conversational AI into diagnostic processes, providing additional support for pathologists.	Evaluate the utility of ChatGPT in supporting cancer diagnosis through AI-driven conversational tools and diagnostic assistance.	AI in Diagnostic Support
			
Slabaugh et al. (2023) [28]	Reviews the application of machine learning and deep learning technologies in thyroid cytology and histopathology. The paper explores how these advanced AI methods enhance diagnostic accuracy and efficiency.	Focuses on the application of machine learning and deep learning in thyroid cytology and histopathology, highlighting improvements in diagnostic processes.	AI in Machine Learning and Deep Learning
			
Lebrun & Salmon (2024) [29]	Provides insights into recent advancements in classifying thyroid neoplasms according to the 2022 WHO classification. It highlights the contribution of AI to improving diagnostic accuracy in this field.	Examines how AI contributes to the diagnosis and classification of thyroid neoplasms, aligning with the updated WHO classification criteria.	AI in Thyroid Neoplasms
			
Singla et al. (2024) [30]	Investigates the role of AI in fungal cytology, focusing on improving the detection and classification of fungal infections. The study emphasizes the future potential of AI in this specialized diagnostic area.	Explores the role of AI in detecting and classifying fungal infections, emphasizing its potential impact on fungal cytology.	AI in Fungal Cytology
			
Sunny et al. (2023) [31]	Discusses the use of CD44-SNA1 markers in cytopathology for identifying high-grade dysplastic and neoplastic oral lesions. The paper evaluates the role of AI in analyzing these markers to enhance diagnostic precision.	Focuses on the application of AI in analyzing CD44-SNA1 markers for oral lesions, aiming to enhance diagnostic accuracy for high-grade dysplastic and neoplastic lesions.	AI in Oral Lesion Diagnosis
			
Wong et al. (2023) [32]	Reviews advancements and current applications of machine learning in thyroid cytopathology. The paper provides an overview of how machine learning improves diagnostic practices in this field.	Provides an overview of machine learning applications in thyroid cytopathology, highlighting advancements and current uses.	AI in Thyroid Cytopathology
			
Ludwig et al. (2023) [33]	Updates on the use of AI in diagnosing and classifying thyroid nodules. The study discusses recent advancements in AI technologies and their integration into diagnostic practices for thyroid conditions.	Highlights advancements in AI for diagnosing and classifying thyroid nodules, reflecting recent developments in the field.	AI in Thyroid Nodule Diagnosis
			
Marletta et al. (2023) [34]	Reviews AI-based tools for diagnosing microbiological diseases, discussing how these technologies improve diagnostic accuracy and efficiency. The paper highlights the impact of AI on pathological diagnoses in microbiology.	Investigates AI tools for diagnosing microbiological diseases, focusing on their impact on pathological diagnoses.	AI in Microbiological Pathology
			
Tessler et al. (2023) [35]	The study reviews the role of AI in diagnosing thyroid nodules, highlighting its potential for improving risk stratification and diagnostic accuracy. It does not cover microbiological diseases.	Reiterates the role of AI in thyroid cytopathology, highlighting advancements and current uses.	AI in thyroid Nodule Diagnosis
			
Hameed & Krishnan (2022) [36]	Examines the application of AI in diagnosing pancreatic cancer, highlighting its benefits, challenges, and the effectiveness of AI-driven diagnostic tools.	Discusses the potential of AI in enhancing pancreatic cancer diagnosis, including the benefits and challenges of AI-driven tools.	AI in Pancreatic Cancer Diagnosis
			
Thakur et al. (2022) [37]	Reviews AI applications in non-gynecological cancer cytopathology, focusing on recent advancements and their impact on diagnostic practices. The study provides a systematic review of the role of AI in this area.	Focuses on AI applications in non-gynecological cancer cytopathology, providing a systematic review of advancements and impacts.	AI in Non-Gynecological Cancer Diagnosis
			
Alrafiah (2022) [38]	Analyzes the application and performance of AI technologies in cytopathology. The study evaluates how different AI technologies enhance diagnostic accuracy and efficiency in the field.	Examines the effectiveness and impact of AI technologies in cytopathology, highlighting their role in improving diagnostic performance.	AI in Cytopathology Performance
			
Vaickus et al. (2024) [39]	Overview of AI advancements in cytopathology, including consensus rule sets such as Bethesda and Paris.	AI offers reproducible and objective diagnoses, addressing variability and biases in human interpretation.	AI in cytopathology standardization
			
Jorda et al. (2024) [40]	Current and future impacts of urinary tract cytopathology on patient care, including AI advancements.	AI enhances diagnostic accuracy and management strategies in urinary tract cytopathology.	AI in urinary tract cytopathology
			
Velez Torres et al. (2024) [41]	Review of AI applications in thyroid fine-needle aspiration (FNA) for improved diagnostic accuracy.	AI enhances diagnostic precision and risk stratification in thyroid FNA cytology.	AI in thyroid cytology

Table 3 presents the key areas of interest. It should be noted that some studies may cover multiple areas; thus, the areas where the contribution appears most dominant have been indicated. Therefore, the table categorizes studies based on their predominant focus areas, highlighting how AI technologies are integrated into various diagnostic processes to enhance accuracy, efficiency, and patient care.

##### AI in Diagnostic Automation and Digital Cytology

The integration of AI into diagnostic automation and digital cytology is transforming traditional methods by improving accuracy and workflow efficiency. Ciaparrone et al. (2024) [21] investigate the role of computer-assisted diagnosis (CAD) systems in urine cytopathology, focusing on their potential to enhance the diagnosis of urothelial carcinomas. Kim et al. (2024) [25,26] review the adoption of digital cytology and AI integration, discussing the benefits and challenges of incorporating these technologies into routine practice.

##### AI in Cancer Diagnosis and Prognosis

In cancer diagnosis and prognosis, AI technologies provide significant advancements by enhancing early detection and diagnostic support. Zhang et al. (2024) [22] explore AI applications in the early diagnosis of thyroid cancer, utilizing ultrasound images and cytopathology samples. Malik & Zaheer (2024) [27] examine the role of ChatGPT in aiding cancer diagnosis, while Slabaugh et al. (2023) [28] review the application of machine learning and deep learning in thyroid cytology and histopathology. Tessler et al. (2022) [35] focus on the role of AI in evaluating thyroid nodules, highlighting its impact on diagnostic accuracy. The application of AI in non-gynecological cancer cytopathology aims to improve diagnostic processes through advanced models. Thakur et al. (2022) [37] evaluate AI applications across various cancer types, focusing on the need for improved dataset quality and validation.

##### AI in Integrated Diagnostics

AI is increasingly integrated into established diagnostic frameworks, enhancing the precision of various diagnostic tools. Caputo et al. (2024) [23], for example, discuss how AI complements molecular and computational advancements in cytopathology. Giovanella et al. (2024) [24] review the integration of AI with multiple diagnostic tools for evaluating thyroid nodules, emphasizing a multimodal approach.

##### AI in Specialized Fields

AI applications are expanding into specialized diagnostic areas such as fungal infections [30] and oral lesions [31], providing innovative solutions for detecting and analyzing these conditions more accurately.

##### AI in Microbiological and Pancreatic Pathology

AI is transforming the diagnosis of microbiological and pancreatic diseases by enhancing diagnostic capabilities and addressing the unique challenges these fields present, leading to more precise and efficient diagnoses [34,36]

##### AI Performance and Challenges

Understanding the performance of AI and addressing its challenges is crucial for its successful implementation in diagnostic settings. Lebrun & Salmon (2024) [29] review updates from the WHO classification of thyroid neoplasms and the role of AI in refining diagnosis. Alrafiah (2022) [38] provides a comprehensive overview of AI advancements in cytopathology, addressing its challenges and future research directions. Vaikus et al. (2024) [39] discuss the integration of AI into cytopathology and its potential to enhance diagnostic consistency.

##### AI in Urinary Tract and Thyroid Cytopathology

In urinary tract cytopathology, AI is enhancing diagnostic accuracy and efficiency. Jorda et al. (2024) [40] discuss the role of AI in urine cytology, addressing challenges related to diagnostic accuracy and false results. AI technologies are making strides in thyroid cytology, improving diagnostic accuracy and risk assessment. Torres et al. (2024) [41] review the integration of AI in thyroid cytology, focusing on its impact on diagnosing thyroid nodules and stratifying risk.

**Table 3:** AI in Cytopathology: Key Areas and Diagnostic Innovations.

Broad Area	References	Overview
AI in Diagnostic Automation and Digital Cytology	[21] Ciaparrone et al. (2024) [25] Kim et al. (2024, Part 2) [26] Kim et al. (2024, Part 1).	AI is enhancing diagnostic efficiency by automating processes and advancing digital cytology, which offers significant improvements in diagnostic speed and cost-effectiveness.
		
AI in Cancer Diagnosis and Prognosis	[22] Zhang et al. (2024) [27] Malik & Zaheer (2024) [28] Slabaugh et al. (2023) [37] Thakur et al. (2022) [35] Tessler et al. (2022)	AI technologies are crucial for advancing cancer diagnosis and prognosis, providing sophisticated tools for early cancer detection, personalized diagnostic support, and better management of cancer cases.
		
AI in Integrated Diagnostics	[23] Caputo et al. (2024) [24] Giovanella et al. (2024) [32] Wong et al. (2023) [33] Ludwig et al. (2023)	AI is integrated into existing diagnostic frameworks to enhance the evaluation of thyroid conditions and other diagnoses, improving the precision of diagnostic results and comprehensive patient assessment.
		
AI in Specialized Fields	[30] Singla et al. (2024) [31] Sunny et al. (2023)	AI applications are expanding into specialized diagnostic areas such as fungal infections and oral lesions, providing innovative solutions for detecting and analyzing these conditions more accurately.
		
AI in Microbiological and Pancreatic Pathology	[34] Marletta et al. (2023) [36] Hameed & Krishnan (2022)	AI is transforming the diagnosis of microbiological and pancreatic diseases by enhancing diagnostic capabilities and tackling the unique challenges these fields present, leading to more precise and efficient diagnoses
		
AI Performance and Challenges	[38] Alrafiah (2022) [29] Lebrun & Salmon (2024) [39] Vaikus et al. (2024)	Analyzes the effectiveness and limitations of AI applications, focusing on how AI improves diagnostic performance while also addressing the challenges and areas needing further development and the perspectives of standardization
		
AI in Urinary Tract and Thyroid Cytopathology	[40] Jorda et al. (2024) [41] Torres et al. (2024)	Highlights the role of AI in urinary tract cytopathology, enhancing diagnostic accuracy. Reviews AI integration in thyroid FNA cytology for better diagnosis and risk stratification.

#### 3.3.2. Opportunities and Areas Needing Broader Investigation

The application of artificial intelligence (AI) in diagnostic practices, particularly within cytopathology, is rapidly advancing, presenting significant opportunities to enhance diagnostic accuracy, efficiency, and workflow management. AI technologies, such as machine learning (ML) and deep learning (DL), are making substantial impacts across various diagnostic domains. For instance, Ciaparrone et al. (2024) [21] highlight the role of computer-assisted diagnosis (CAD) systems in improving the diagnosis of urothelial carcinomas by identifying subtle patterns that human reviewers might miss. Similarly, Zhang et al. (2024) [22] discuss the transformative potential of AI in the early diagnosis and risk stratification of thyroid cancer through the analysis of imaging and molecular data.

Opportunities are extensive, with AI tools enhancing diagnostic processes and offering new insights into complex cases. Kim et al. (2024) [25,26] provide a comprehensive review of the integration of digital cytology and AI, recommending best practices for improving diagnostic accuracy and laboratory workflows. Malik and Zaheer (2024) [27] explore how ChatGPT can support cancer diagnosis by processing large volumes of data and providing additional analytical perspectives.

Despite these advancements, several areas require further investigation. Slabaugh et al. (2023) [28] emphasize the need for prospective validation of ML and DL algorithms in thyroid cytology and histopathology, addressing issues related to algorithm interpretability and clinical integration. Giovanella et al. (2024) [24] call for more research to establish the clinical value of AI when combined with other diagnostic tools for thyroid nodules. Marletta et al. (2023) [34] stress the need for technological improvements and better datasets to expand the adoption of AI in microbiological diagnostics.

Challenges also persist, such as ensuring the practical usability and cost-effectiveness of AI tools. Wong et al. (2023) [32] highlight the necessity for diverse datasets and further validation to refine ML algorithms for thyroid cytopathology. Jorda et al. (2024) [40] note the importance of correlating urine cytology findings with tissue samples to minimize false results. Tessler et al. (2022) [35] discuss the need to address the practical usability and cost-effectiveness of AI tools in clinical settings.

The following table summarizes the key studies, outlining both the significant opportunities presented by AI in diagnostic practices and the areas that require further exploration to address existing challenges and ensure effective integration into clinical settings. Table 4 reports the emerging opportunities and the areas needing a broader investigation.

**Table 4:** Emerging opportunities and areas needing a broader investigation.

Study	Opportunities	Areas Needing Broader Investigation
Ciaparrone et al. (2024) [21]	Enhances diagnosis of urothelial carcinomas with CAD systems, improving diagnostic accuracy and workflow efficiency.	Requires rigorous validation and regulatory approval. Comprehensive training for pathologists is needed.
		
Zhang et al. (2024) [22]	Improves early diagnosis and risk stratification of thyroid cancer through AI analysis of ultrasound images and molecular markers.	Further development and clinical validation are necessary. Expansion of AI tools in routine practice is needed.
		
Caputo et al. (2024) [23]	Integrates digital pathology and molecular advancements for precise cancer risk stratification. Enhances diagnostic accuracy and cost-effectiveness.	Need for effective integration of digital and AI technologies in clinical settings. Perspectives on new tools from pathologists require exploration.
		
Giovanella et al. (2024) [24]	Combines ultrasound, FNAC, molecular imaging, and AI to refine the diagnosis of thyroid nodules and reduce unnecessary procedures.	Further research is needed to establish the clinical value of AI and its effectiveness in combination with other diagnostic tools.
		
Kim et al. (2024) [25,26]	Reviews and guides the integration of digital cytology and AI into cytology workflows, improving diagnostic accuracy and efficiency. Provides best practice recommendations.	Challenges include technology costs, workflow integration, standardized protocols, and the need for effective implementation strategies.
		
Malik and Zaheer (2024) [27]	Enhances cancer diagnosis by integrating ChatGPT and digital slides for additional analysis and knowledge synthesis.	Challenges include integrating AI with existing systems, addressing biases, and navigating legal issues.
		
Slabaugh et al. (2023) [28]	ML and DL technologies address limitations in thyroid cytology and histopathology, improving the classification and diagnosis of thyroid lesions.	Need for prospective validation, improving algorithm interpretability, and integrating into clinical workflows.
		
Lebrun and Salmon (2024) [29]	Updates in thyroid neoplasm classification and molecular testing improve diagnosis and risk stratification.	Ongoing challenges in managing low-risk lesions and integrating AI into diagnostic strategies.
		
Singla et al. (2024) [30]	Transforms detection and typing of fungal infections using AI technologies, improving accuracy and real-time identification.	Requires further research to explore the full potential of AI in fungal cytology.
		
Sunny et al. (2023) [31]	Integrates biomarkers with AI to enhance diagnostic sensitivity and specificity for oral potentially malignant disorders.	Further research is needed to refine biomarker panels and automate image analysis for point-of-care diagnostics.
		
Wong et al. (2023) [32]	Promises improved diagnostic accuracy and efficiency in thyroid cytopathology through ML integration.	Need for larger, diverse datasets and further validation studies to refine ML algorithms and clinical integration.
		
Ludwig et al. (2023) [33]	AI improves the classification and management of thyroid nodules, potentially reducing unnecessary procedures.	Ongoing research is required to validate AI tools and enhance clinical applicability.
		
Marletta et al. (2023) [34]	Enhances microbiological disease diagnosis, particularly in resource-limited settings, by analyzing cytological images with AI.	Technological improvements and better datasets are needed to expand the adoption of AI in microbiological diagnostics.
		
Tessler et al. (2022) [35]	Utilizes AI for evaluating thyroid nodules, enhancing diagnostic accuracy and efficiency, particularly benefiting less experienced physicians.	Challenges include ensuring the practical usability and cost-effectiveness of AI tools in clinical settings.
		
Thakur et al. (2022) [37]	AI shows potential in enhancing non-gynecological cancer diagnostics, with promising results in various cancer types.	Requires larger, well-annotated datasets and external validation to improve AI models and their clinical application.
		
Alrafiah et al. (2022) [38]	Provides a comprehensive overview of AI advancements in cytopathology, highlighting improvements in diagnostic accuracy and workflow.	Emphasizes the need for transparency, robust validation, and practical integration into clinical workflows.
		
Vaikus et al. (2023) [39]	AI integration with consensus rule sets aims to reduce diagnostic variability and enhance accuracy.	Requires addressing biases and variability in AI systems to realize its potential in cytopathology fully.
		
Jorda et al. (2024) [40]	Enhances urinary tract cytopathology with AI, improving diagnostic accuracy and patient management.	Need for improved correlation between cytology findings and tissue samples to minimize false results.
		
Torres et al. (2024) [41]	Integrates AI with FNA and molecular testing in thyroid cytology, improving diagnostic accuracy and risk stratification.	Challenges include integrating AI with traditional methods and ensuring comprehensive validation in clinical settings.

#### 3.3.3. Integrating AI into Cytopathology: Recommendations for Advancing Diagnostic Practices

The integration of artificial intelligence (AI) into cytopathology is transforming diagnostic practices by enhancing accuracy, efficiency, and workflow. To fully harness the benefits of AI, several key recommendations have emerged from the overview of reviews, as summarized in Table 4:

Further Research and Validation: It is crucial to continue refining AI algorithms, expanding datasets, and conducting prospective validation to address current limitations and improve AI performance [21,22,28].

Integration into Clinical Practice: Effective implementation of AI tools involves addressing practical challenges such as usability, system interoperability, and seamless incorporation into routine workflows [23,25,27].

Training and Education: Comprehensive training for pathologists and laboratory staff is essential to use AI tools effectively, understand their capabilities and limitations, and integrate them into daily practices [24,25,41].

Regulatory and Ethical Considerations: Rigorous validation and regulatory approval are necessary to ensure AI tools meet clinical standards and ethical guidelines, preventing biases and ensuring equitable diagnostic practices [21,27,38].

Combining AI with Existing Methods: Leveraging AI alongside traditional methods and molecular testing can enhance diagnostic accuracy and provide a more comprehensive assessment [23,24,33].

Addressing Cost and Infrastructure: Consideration of technology costs and the establishment of standardized protocols is vital for effective AI integration and widespread adoption [25,32,34].

These recommendations are detailed in Table 5, which provides a summary of the key findings and strategies for advancing AI integration into cytopathology.

**Table 5:** Recommendations for Advancing AI Integration in Cytopathology.

Recommendation	Description	References
Further Research and Validation	Continue refining algorithms, expand datasets, and conduct prospective validation to address limitations and improve performance.	[21,22,28]
		
Integration into Clinical Practice	Ensure AI tools complement existing methods by addressing usability, system interoperability, and workflow incorporation.	[23,25,27]
		
Training and Education	Provide comprehensive training for pathologists and laboratory staff to use AI tools and integrate them into practice effectively.	[24,25,41]
		
Regulatory and Ethical Considerations	Address regulatory approval, validation, and ethical guidelines to ensure AI tools meet clinical standards and prevent biases.	[21,27,38]
		
Combining AI with Existing Methods	Use AI alongside traditional methods and molecular testing to enhance diagnostic accuracy and provide comprehensive assessments.	[23,24,33]
		
Addressing Cost and Infrastructure	Consider technology costs and establish standardized protocols for effective AI integration and widespread adoption.	[25,32,34]

As can be seen in the table, ethics and standardization emerge as cross-cutting themes. For this reason, we have decided to further refine the analysis by focusing on specific recommendations related to these aspects.

Additionally, a fundamental aspect of optimal integration into the health domain is the ongoing effort to generate empirical data demonstrating the performance of AI in diagnostic cytopathology, particularly in terms of diagnostic precision. This is a theme to be addressed primarily in cutting-edge primary studies, as reviews often have a structural delay in forming conclusions because of their foundational data.

The integration of artificial intelligence (AI) into cytopathology presents significant opportunities for enhancing diagnostic accuracy and efficiency. However, to fully realize these benefits, it is essential to establish standardized practices that ensure the reliable and effective use of AI technologies.

This involves addressing several key areas, including validation, data quality, protocol integration, training, performance monitoring, and collaboration.

Validation and benchmarking are crucial for ensuring that AI tools perform consistently across different settings and populations. Standardized validation protocols help in assessing the reliability and accuracy of AI systems, making it possible to compare their performance with existing diagnostic methods and among various AI models [26,32].

Data consistency and quality play a vital role in the development and performance of AI models. Uniform data collection and preprocessing procedures are necessary to ensure that AI systems are trained on high-quality, consistent datasets. This consistency helps in achieving reliable diagnostic results and reducing biases [27,31].

Protocol integration involves incorporating AI tools seamlessly into existing diagnostic workflows. Standardized protocols for AI integration ensure that these tools complement traditional methods effectively, maintaining workflow efficiency and diagnostic accuracy [33,34].

Training and education are essential for pathologists and laboratory technicians to use and interpret AI tools effectively. Comprehensive training programs and educational resources should be developed to equip users with the skills needed to harness AI technologies effectively [30,31].

Performance monitoring is required to maintain the reliability of AI systems over time. Regular evaluations and quality control measures help in identifying and addressing performance issues, ensuring that AI tools continue to deliver accurate and reliable results [38,39].

Collaboration and communication among AI developers, healthcare professionals, and regulatory bodies are crucial for successful AI implementation. Effective communication channels and collaborative efforts help in addressing challenges and ensuring that AI technologies are integrated and regulated appropriately [26,27].

By focusing on these standardization aspects, the cytopathology field can ensure that AI technologies are used effectively, improving diagnostic outcomes and advancing patient care. The following Table 6 outlines specific recommendations for achieving these standards.

**Table 6:** Recommendations focused on the standardization.

Standardization Aspect	Description	Recommendations	References
Validation and Benchmarking	Ensuring AI tools meet consistent performance standards across diverse settings is crucial for reliable diagnostics.	Develop and adhere to standard validation protocols and actively participate in benchmarking studies to compare AI performance across different datasets and settings.	[26,32]
			
Data Consistency and Quality	High-quality, consistent data are essential for training effective AI models and ensuring accurate predictions.	Implement standardized procedures for data collection, annotation, and pre-processing to ensure data uniformity and enhance model training.	[27,31]
			
Protocol Integration	Seamless integration of AI tools into existing diagnostic workflows and protocols is necessary for efficient use.	Establish and follow standardized protocols for incorporating AI tools into clinical practice, ensuring they complement existing diagnostic methods.	[33,34]
			
Training and Education	Pathologists and laboratory technicians need proper training to use AI tools and interpret their outputs effectively.	Develop comprehensive training programs and educational resources to ensure that users of AI technologies are well-prepared to utilize and evaluate AI-assisted diagnostics.	[30,31]
			
Performance Monitoring	Continuous evaluation is necessary to maintain AI system performance and address any emerging issues.	Implement regular performance monitoring and quality control measures and update AI systems as needed to sustain reliability and accuracy over time.	[38,39]
			
Collaboration and Communication	Effective communication between AI developers, healthcare professionals, and regulatory bodies is crucial for successful AI implementation.	Foster communication channels and collaborative efforts among stakeholders to address challenges and ensure the successful integration and regulation of AI technologies.	[26,27]

The integration of artificial intelligence (AI) into cytopathology presents numerous ethical challenges and considerations. As AI technologies advance and become more prevalent in diagnostic processes, addressing these ethical issues is crucial to ensuring that AI applications are used responsibly and equitably. Here’s an overview of the key ethical considerations and recommendations for AI in cytopathology:

Key ethical considerations in AI for cytopathology bias and fairness in AI systems are susceptible to biases present in training data. If data used to train AI models are not representative of diverse populations, the resulting models may exhibit biases that can lead to unequal diagnostic performance across different demographic groups. Ensuring fairness requires using diverse and representative datasets and regularly auditing AI systems for bias [27,34]. Transparency and explainability of the decision-making process of AI systems should be transparent and explainable to users. Pathologists and clinicians need to understand how AI systems arrive at their conclusions to trust and effectively use these tools. Developing AI systems with clear, interpretable models and providing explanations for AI-generated recommendations are essential for maintaining trust in AI-assisted diagnostics [26,27]. Data privacy and security handling of patient data responsibly is paramount. AI systems in cytopathology require access to sensitive patient information, which must be protected from unauthorized access and misuse. Implementing robust data security measures and complying with regulations such as GDPR or HIPAA are critical for safeguarding patient privacy [38,39]. Informed consent: patients should be informed about the use of AI in their diagnostic process. This includes understanding how their data will be used, the role of AI in their diagnosis, and any potential implications. Ensuring that patients provide informed consent before their data are used for AI applications is an important ethical requirement [30,33]. Accountability and responsibility for determining accountability when AI systems make errors or provide misleading results, is essential. It is necessary to establish clear guidelines on the responsibilities of AI developers, healthcare providers, and institutions in managing and addressing potential errors in AI-assisted diagnoses [31,32]. Regulatory compliance: AI systems must comply with relevant regulatory standards and guidelines to ensure their safety and effectiveness. Adhering to regulatory requirements and undergoing rigorous validation processes are vital for maintaining high standards in AI-assisted diagnostics [27,38].

Table 7 provides a comprehensive overview of ethical considerations and recommendations for integrating AI into cytopathology. Addressing these concerns is essential for the responsible and effective implementation of AI technologies in diagnostic practices.

**Table 7:** Ethical Recommendations for AI in Cytopathology.

Ethical Consideration	Description	Recommendations	References
Bias and Fairness	AI models may exhibit biases if trained on non-representative data.	Ensure diverse datasets, conduct regular audits for bias, and implement fairness metrics.	[27,34]
			
Transparency and Explainability	AI decision-making processes should be understandable to users.	Develop explainable AI models and provide clear explanations for AI-generated recommendations.	[26,27]
			
Data Privacy and Security	Protect sensitive patient data from unauthorized access and misuse.	Implement robust data security measures and comply with relevant privacy regulations.	[38,39]
			
Informed Consent	Patients must be aware of and consent to the use of AI in their diagnosis.	Obtain informed consent from patients, including details on AI usage and data handling.	[30,33]
			
Accountability and Responsibility	Define clear accountability for errors or misleading results from AI systems.	Establish guidelines for AI developers, healthcare providers, and institutions regarding AI errors.	[31,32]
			
Regulatory Compliance	Adhere to regulatory standards to ensure AI systems’ safety and effectiveness.	Follow regulatory requirements and validation procedures for AI systems.	[27,38]

## 4. Discussion

The discussion is structured into the following sections:

Section 4.1 reports a synoptic diagram

Section 4.2 presents and discusses the added value of the umbrella review within the specific field.

Section 4.3, based on the recommendations emerging from the umbrella review results, focuses on specific aspects of standardization, ethics, and diagnostic accuracy (with the corresponding need for primary data). This section, organized into subsections, provides a deeper analysis through the additional exploration of primary studies in this area.

Section 4.4, organized into subsections, examines the integration of AI in cytopathology, comparing it with the experiences in histopathology and radiology. The experiences in these two fields, particularly in radiology, offer a potential roadmap for improving AI integration in cytopathology.

Section 4.5 concludes by outlining the limitations of the study.

### 4.1. Synoptic Diagram

**Figure 7:**
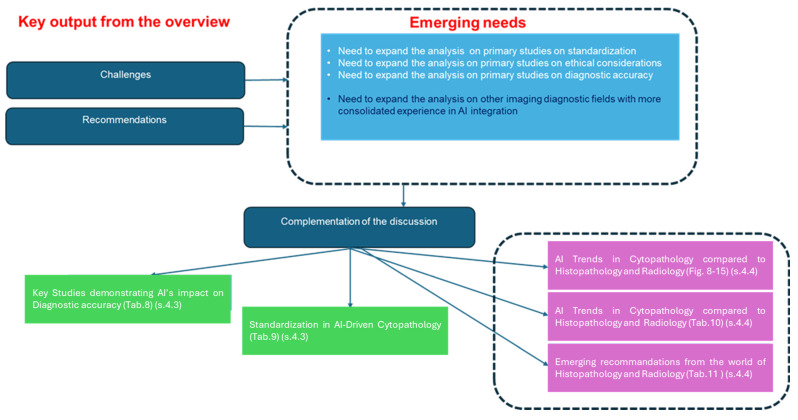
Synoptic diagram reporting a sketch of the discussion.

The diagram in Figure 7 presents a concise overview of the discussion, organized into tabular connections and diagrams in alignment with the overall aim and specific objectives. Based on the findings from the analysis in the results of the umbrella review, specific focal points have emerged for discussion, highlighting recommendations and areas that require targeted exploration and complementation (see also Figure 2).

These focal points are displayed in Block 1 at the top right and address key aspects such as standardization, ethical considerations, and diagnostic accuracy. Additionally, the initial hypotheses have been confirmed, indicating that cytopathology lags histopathology and, particularly, radiology in integrating artificial intelligence (AI). This has underscored the need for a comparative analysis. Complementation in this regard has been pursued, as illustrated in Block 2 (descending from top to bottom). This part of the discussion has generated significant insights regarding primary studies on standardization and diagnostic accuracy, as summarized in Block 3 (moving down left) and Block 4 (moving right), which synthesize evidence from primary studies in these areas in Table 7 and Table 8.

**Figure 8:**
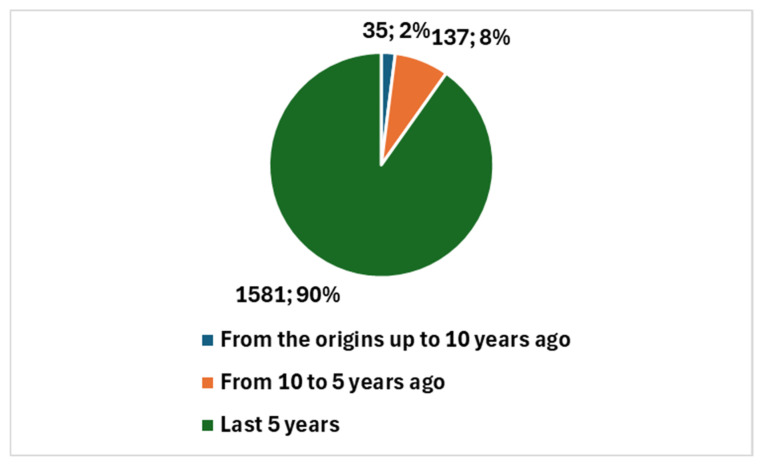
Temporal trends in scientific production in histopathology &AI with details for the last five and ten years.

**Figure 9:**
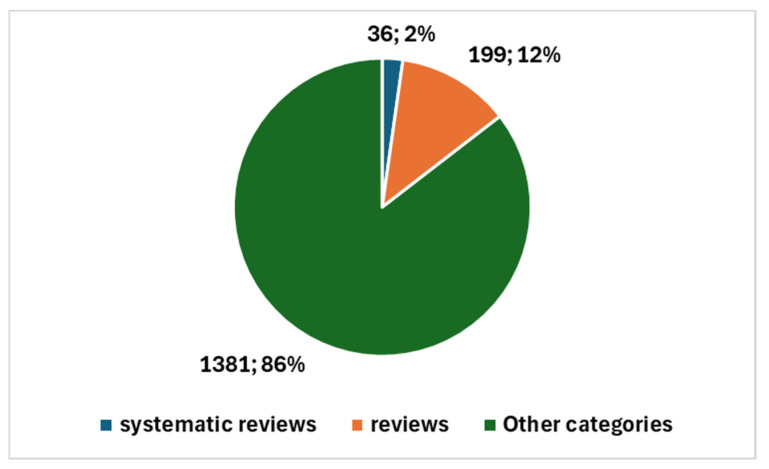
Scientific production in histopathology & AI (reviews and systematic reviews are illustrated separately.

**Figure 10:**
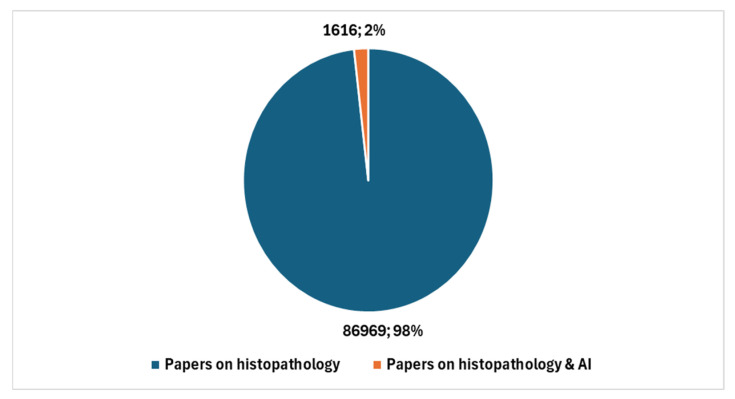
Ratio of AI-Focused to Non-AI-Focused Research in Histopathology.

**Figure 11:**
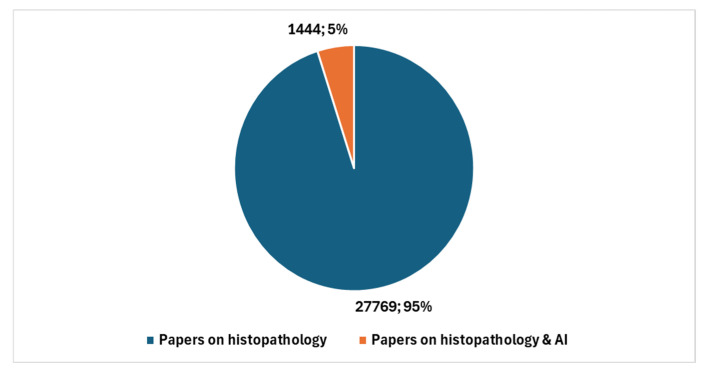
Ratio of AI-Focused to Non-AI-Focused Research in Histopathology in last five years.

**Figure 12:**
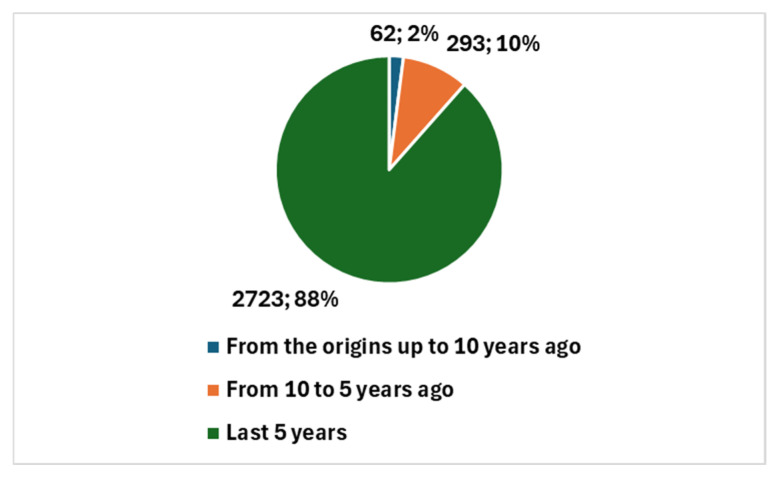
Temporal trends in scientific production in radiology & AI with details for the last five and ten years.

**Figure 13:**
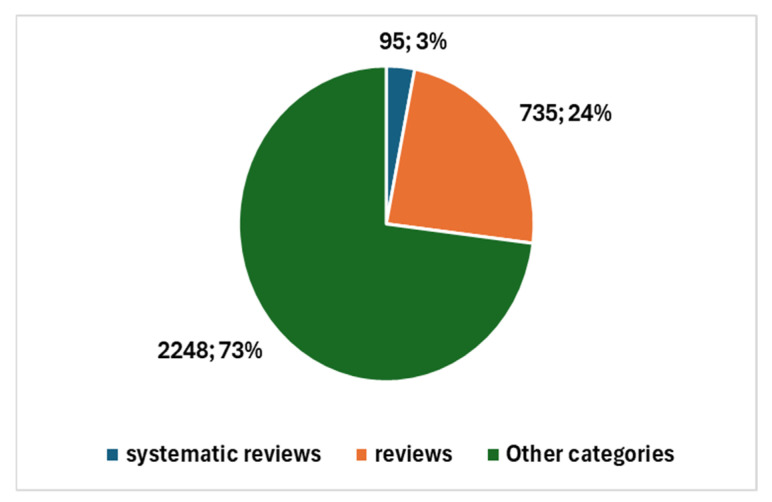
Scientific production in radiology & AI (reviews and systematic reviews are illustrated).

**Figure 14:**
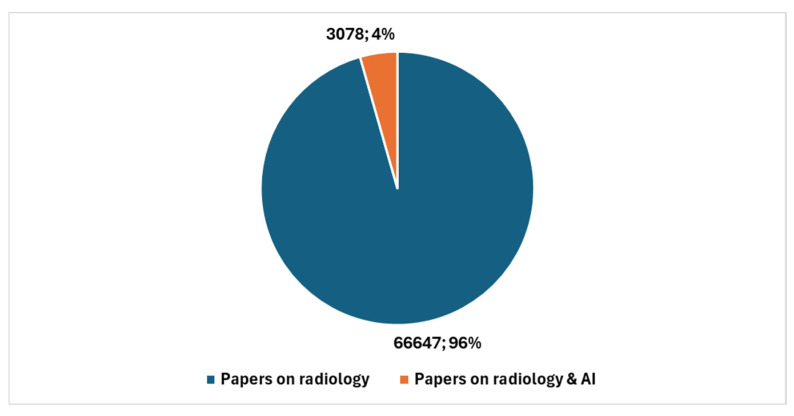
Ratio of AI-Focused to Non-AI-Focused Research in radiology.

The group of blocks on the right (Blocks 5–7) conducts a comparison of the situations in radiology and histology, graphically presenting trends (Figure 7, Figure 8, Figure 9, Figure 10, Figure 11, Figure 12, Figure 13 and Figure 14 in Block 5), a comparative analysis of scientific output volume (Table 9 in Block 6), and the recommendations emerging from the domains of radiology and histopathology aimed at cytopathology for better integration with AI.

### 4.2. The Umbrella Review: Added Value and Highlights

An increasing interest from professionals in the integration of AI in cytopathology is being observed [9].

An umbrella review in the realm of cytopathology and artificial intelligence (AI) is crucial for synthesizing the diverse and rapidly expanding body of research on this intersection. The significance of such a review lies in its ability to collate findings from various reviews, offering a comprehensive overview of the current advancements and challenges in this field.

As AI continues to enhance diagnostic accuracy and efficiency in cytopathology, it is vital to address emerging themes such as integration challenges, bias, and cost-effectiveness. By consolidating evidence from multiple studies, an umbrella review not only highlights the practical and theoretical gaps but also provides actionable insights for improving the implementation of AI technologies. This holistic perspective supports informed decision-making and fosters the development of best practices, ultimately guiding the effective integration of AI tools into clinical workflows and advancing the overall quality of diagnostic cytopathology. The umbrella review underscores the profound impact of artificial intelligence (AI) on diagnostic cytopathology, emphasizing its role in enhancing both the speed and accuracy of diagnostic processes. By integrating AI technologies, traditional methods of image analysis and diagnostic evaluation are significantly improved, leading to more efficient workflows and reduced diagnostic errors [21,22,23,24,25,26]. The ability of AI to automate and optimize image analysis is particularly notable in areas such as urine cytology and thyroid pathology, where it has demonstrated substantial benefits in early cancer detection and risk assessment [21,22,24,35].

Recent studies have shown that AI applications are not only accelerating diagnostic procedures but are also refining the precision of results through advanced machine learning models and digital cytology practices [27,32]. This is evidenced by the integration of AI into various diagnostic frameworks, such as those used for evaluating thyroid nodules and urinary tract conditions [24,40]. Despite these advancements, challenges related to standardization and the need for continuous validation of AI tools remain critical areas for ongoing research [38,39].

Overall, this review illustrates how AI can contribute to advancing diagnostic practices by enhancing accuracy and efficiency. It also highlights the ongoing need for further research to address the challenges and optimize AI integration in clinical settings [41].

The recommendations emerging from the umbrella review highlight key areas for advancing AI integration in cytopathology. Ethical considerations and the need for standardization are crucial, emphasizing the importance of ensuring that AI tools meet clinical standards and avoid biases. Establishing standardized practices, including uniform validation protocols, is essential to maintain diagnostic reliability across various settings [21,27,38]. Additionally, the generation of empirical data demonstrating the diagnostic accuracy of AI is fundamental. Ongoing research is required to validate AI performance in clinical environments, ensuring that tools are both effective and reliable [21,22,28].

This comprehensive analysis provides valuable insights, based on consolidated them, into the current state and future potential of AI in cytopathology, making a strong case for its continued development and application [21,22,23,24,25,26,27,28,29,30,31,32,33,34,35,36,37,38,39,40,41]

### 4.3. Deepening the Analysis: Exploring New Dimensions and Implications from Cutting-Edge Research

Among the cross-cutting recommendations emerging from the reviews across various studies are two primary areas of focus: (a) advancing towards specific and targeted research to produce empirical data, particularly in the promising area of diagnostic accuracy and AI related to cytopathology, and (b) concentrating on standardization, a crucial aspect for integrating methodologies into stable routines within the health domain.

Empirical data, which refer to information gathered through direct observation, experimentation, or measurement, are essential for validating the effectiveness and practical applicability of AI systems in cytopathology. Such data are derived from real-world studies that provide concrete evidence on the performance, reliability, and clinical integration of AI tools in diagnostic tasks.

To address these recommendations, the umbrella review specifically targeted these two topics, as reflected in primary studies (non-review studies but articles, comparative studies, etc.). These studies include:

Diagnostic Accuracy in AI for Cytopathology: Selected studies based on a composite key in Box 2, Position 1, include [42,43,44,45,46,47,48,49].

Standardization: As a critical component for integrating methodologies into routine health practices, we reviewed studies addressing this aspect using selected studies based on composite keys in Box 2, Position 2, including [50,51,52,53,54,55].

In the Supplementary Materials there is also a detailed analytical summary of each one of these studies [42,43,44,45,46,47,48,49,50,51,52,53,54,55].

By focusing on these studies, we aim to provide a comprehensive integrative and differential evaluation of the current state of diagnostic accuracy and standardization in AI applications for cytopathology. It is crucial to emphasize that many of the cross-cutting recommendations in the literature not only touch on technical and clinical aspects but also bring attention to the ethical considerations surrounding the use of AI in healthcare. Ethics plays a pivotal role, especially in areas where AI technologies intersect with sensitive decision-making processes, such as diagnostics, treatment planning, and patient privacy. Given the profound implications of AI-driven systems in medical settings, exploring ethical dimensions becomes indispensable.

However, a focused exploration of primary studies on ethics reveals a noticeable gap. Our search, based on a composite key (outlined in Box 2, Position 3), showed a surprising absence of primary studies explicitly directly focused on ethical issues in AI implementation, particularly within specific domains such as diagnostic cytopathology or patient data privacy in AI applications.

This result is significant, as it suggests that while ethical concerns are frequently highlighted in broader discussions or the recommendation sections of review articles, they are not receiving the focused empirical investigation they warrant. Most existing studies tend to address ethical concerns indirectly, subsuming them under general technological or clinical outcomes without placing them at the forefront of analysis. This indirect treatment leaves a gap in the development of specific ethical frameworks or guidelines that could help navigate the challenges AI poses in healthcare.

The absence of primary studies with a dedicated, ethical focus points to a need for research that not only outlines these concerns in theoretical terms but also grounds them in practical, real-world scenarios. With AI continuing to evolve and integrate into healthcare systems, the ethical implications—such as the transparency of AI decision-making, accountability, fairness, and patient consent—need to be explored through dedicated primary studies. These studies would provide concrete data and insights that could help shape more robust, ethically informed AI applications.

Box 2 **The proposed composite keys in the analysis.**

*(Cytopathology [Title/Abstract]) AND ((Artificial intelligence [Title/Abstract]) OR (machine learning [Title/Abstract]) OR (deep learning [Title/Abstract]) OR (neural network [Title/Abstract])) AND (diagnostic accuracy [Title/Abstract])*

*(Cytopathology [Title/Abstract]) AND ((Artificial intelligence [Title/Abstract]) OR (machine learning [Title/Abstract]) OR (deep learning [Title/Abstract]) OR (neural network [Title/Abstract])) AND ((standard [Title/Abstract) OR (regulation [Title/Abstract))*

*(Cytopathology [Title/Abstract]) AND ((Artificial intelligence [Title/Abstract]) OR (machine learning [Title/Abstract]) OR (deep learning [Title/Abstract]) OR (neural network [Title/Abstract])) AND (ethics [Title/Abstract))*

#### 4.3.1. Deepening the Analysis: Exploring New Dimensions and Implications from Cutting-Edge Research on Diagnostic Accuracy in Cytopathology

Artificial intelligence (AI) is revolutionizing the field of cytopathology by offering advanced tools that significantly enhance diagnostic accuracy. Technologies such as deep learning models and convolutional neural networks have demonstrated considerable potential in various diagnostic domains, including urine cytology, thyroid cytology, and breast cancer diagnostics [42,43,44,45,46,47,48,49]. However, to fully realize these benefits, AI integration into clinical practice necessitates a robust foundation of empirical evidence to validate its efficacy across diverse clinical environments and patient populations.

The importance of fresh empirical data cannot be overstated. Many existing studies are retrospective or involve small, non-representative datasets, which may not fully capture clinical variability. Therefore, large-scale, prospective studies are essential for evaluating the performance of AI in real-world settings and its impact on diagnostic accuracy and patient outcomes. This is crucial for ensuring that AI tools are reliable and effective across different patient populations [42,43,44,45,46,47,48,49].

Recent research highlights the promising role of AI in improving diagnostic accuracy in cytopathology. For instance, Liu et al. [42] found that the AIxURO platform markedly improved sensitivity and efficiency in bladder cancer diagnosis through urine cytology. Zhao et al. [43] demonstrated that the ResNeSt model effectively differentiated between papillary thyroid carcinoma and benign nodules. Mhaske et al. [44] explored machine learning algorithms for analyzing nuclear features in oral exfoliative cytology, showcasing their potential to enhance diagnostic precision. Similarly, Kim et al. [45] and Park et al. [46] provided evidence of AI models significantly improving diagnostic accuracy for lung cancer and metastatic breast cancer, respectively.

Moreover, integrating AI into cytopathology presents both opportunities and challenges that require further investigation. Recent studies have shown the efficacy of advanced AI technologies in various contexts. For example, Ozer et al. [47] demonstrated that a deep neural network achieved 95% diagnostic accuracy for brain tumors in intraoperative cytology, while Saikia et al. [48] found that GoogLeNet-V3 achieved 96.25% accuracy in classifying breast FNAC images. Sanyal et al. [49] also highlighted the potential of AI in thyroid FNAC smears, showing 85.06% diagnostic accuracy for distinguishing between papillary and non-papillary carcinoma.

Table 8 provides a detailed summary of these empirical studies, illustrating the ongoing efforts to refine AI technologies to support diagnostic accuracy in cytopathology. This table underscores the need for additional empirical evidence and the importance of addressing challenges related to data quality, protocol integration, training, performance monitoring, and collaboration [47,48,49].

**Table 8:** Key Studies Demonstrating the Impact of AI on Diagnostic Accuracy.

Study	AI Tool/Model	Application Area	Key Findings	References
Liu TJ et al. [42]	AIxURO	Urine Cytology for Bladder Cancer	AIxURO improved sensitivity from 25.0–63.9%, positive predictive value (PPV) from 21.6–31.1%, and reduced screening time by 52.3–83.2%.	[42]
				
Zhao D et al. [43]	ResNeSt	Thyroid Nodules Diagnosed as AUS	Achieved 92.49% accuracy and improved sensitivity (95.79%) and specificity (88.46%) in differentiating papillary thyroid carcinoma from benign nodules.	[43]
				
Mhaske S et al. [44]	CNN vs. SVM	Oral Exfoliative Cytology	The study found substantial variations between the study and control groups in nuclear size (*p* < 0.05), nuclear shape (*p* < 0.01), and chromatin distribution (*p* < 0.001). The Pearson correlation coefficient of SVM was 0.6472, and CNN was 0.7790, showing that SVM had more accuracy.	[44]
				
Kim T et al. [45]	Densenet121	Lung Cancer	Increased sensitivity to 95.9% and specificity to 98.2% compared with pathologists; enhanced interobserver agreement (Fleiss’ Kappa from 0.553 to 0.908).	[45]
				
Park HS et al. [46]	Inception-ResNet-V2	Metastatic Breast Cancer in Pleural Fluid	Outperformed pathologists with an accuracy of 81.1%, sensitivity of 95.0%, and specificity of 98.6%; improved pathologists’ diagnostic metrics after AI assistance.	[46]
				
Ozer E et al. [47]	Deep Neural Network	Brain Tumors (Intraoperative Cytology)	Achieved 95% diagnostic accuracy in patch-level classification and 97% at the patient-level classification for brain tumors.	[47]
				
Saikia AR et al. [48]	Various CNN Architectures	Breast FNAC Images	GoogLeNet-V3 achieved 96.25% accuracy in classifying benign and malignant breast FNAC images, showing improved diagnostic reliability.	[48]
				
Sanyal P et al. [49]	Properly proposed and customized Artificial Neural Network	Thyroid FNAC Smears	Demonstrated 85.06% diagnostic accuracy for distinguishing papillary carcinoma from non-papillary carcinoma thyroid lesions.	[49]

Overall, the table provides a summary sustained by a sketch of empirical data of significant studies that highlight the role of AI in improving diagnostic accuracy across various cytopathological contexts. These studies illustrate the effectiveness of AI tools in enhancing diagnostic precision, reducing interobserver variability, and improving overall efficiency.

In conclusion, while AI offers substantial advancements in cytopathology, ongoing empirical research is essential to validate its effectiveness. Such research will ensure that AI technologies not only perform well in controlled environments but also offer practical benefits in diverse clinical settings. As the field evolves, continued empirical validation will be crucial for achieving widespread, reliable integration of AI into routine cytopathological practice.

#### 4.3.2. Deepening the Analysis: Exploring New Dimensions and Implications from Cutting-Edge Research on Standardization

In recent years, the rapid advancement of diagnostic technologies has underscored the importance of standardization to ensure accuracy and reproducibility across various applications. As new methodologies are introduced and refined, issues related to sample preparation, technology integration, and image processing become increasingly prominent. These challenges are central to achieving reliable and consistent diagnostic results.

The following Table 9 provides an overview of key studies that address different aspects of standardization in diagnostic technologies with AI in cytopathology. Each study highlights specific standardization issues and their implications, shedding light on how uniform procedures and protocols can enhance diagnostic practices. The studies included are reported in ref. [50,51,52,53,54,55,56]. In brief (an analytical summary is reported in the Supplementary Materials for each study). In brief:

Peñaranda et al. (2018) [50] explored the impact of sample preparation on Fourier transform infrared (FTIR) spectroscopy, emphasizing the need for consistency to achieve reliable spectral results.

Kumar et al. (2020) [51] reviewed whole-slide imaging (WSI) technologies, focusing on the integration of digital scanning, image visualization, and AI algorithms and the necessity of standardized protocols for effective implementation.

Liu et al. (2022) [52] investigated the effects of cell aspect ratio on deep learning model performance in cytopathology, discussing standardized preprocessing techniques to ensure consistent image dimensions.

Chen et al. (2021) [53] proposed a framework for automatic WSI diagnosis, highlighting the importance of standardized methods for unit selection and attention fusion to improve diagnostic accuracy.

Zhou et al. (2024) [54] compared the performance of AI diagnostic systems with traditional fine needle aspiration (FNA) cytopathology for thyroid nodules, stressing the need for standardized thresholds in AI systems to ensure comparable performance.

Sohn et al. (2023) [55] introduced a deep-learning model for pancreatic cancer diagnosis and discussed the importance of standardized training and evaluation procedures to enhance model reliability.

The table below summarizes these studies, illustrating the critical aspects of standardization and their impact on diagnostic technology:

**Table 9:** Standardization in AI-Driven Cytopathology: Insights from Emerging Diagnostic Technologies.

Study	Focus	Standardization Aspect	Description	Key Findings
Peñaranda et al. (2018) [50]	FTIR Spectroscopy	Sample Preparation	Focuses on the consistency of sample preparation across different batches. Variations in preparation methods can significantly impact spectral results and classification accuracy.	Variability in sample preparation affects classification accuracy. Standardizing sample preparation methods is crucial for reliable results.
				
Kumar et al. (2020) [51]	Whole-Slide Imaging (WSI)	Technology Integration and Protocols	Examines the integration of WSI technology into routine pathology practice, including digital scanning, image visualization, and AI algorithms. Addresses the need for standardized protocols for successful implementation.	Standardization in WSI technology and protocols could address high costs and technical challenges, facilitating broader adoption and consistency in diagnostic practices.
				
Liu et al. (2022) [52]	Deep Learning and Aspect Ratio	Image Preprocessing and Resizing	Investigates the impact of cell aspect ratio on deep learning model performance, focusing on preprocessing techniques to standardize image dimensions without compromising diagnostic quality.	Deep learning models are robust to changes in aspect ratio, suggesting that standardized preprocessing techniques can maintain model performance across varied images.
				
Chen et al. (2021) [53]	Automatic WSI Diagnosis	Selection and Fusion Techniques	Develops a framework for selecting and fusing image units (e.g., patches or cells) for diagnosis. Emphasizes the need for standardized methods in unit selection and fusion to ensure consistent and accurate diagnoses.	Standardization in unit selection and attention fusion improves diagnostic consistency and accuracy across different types of slide images.
				
Zhou et al. (2024) [54]	AI vs. FNA for Thyroid Nodules	AI Diagnostic Systems and Thresholds	Compares AI diagnostic systems with traditional FNA cytopathology and mutation analysis, highlighting the importance of standardized thresholds for AI system performance.	Standardizing AI diagnostic tools can provide performance comparable to traditional methods, potentially enhancing efficiency and reducing the need for invasive procedures.
				
Sohn et al. (2023) [55]	Deep Learning for Pancreatic Cancer	Model Training and Evaluation	Proposes a deep learning model for pancreatic cancer diagnosis and evaluates its performance against existing models. Emphasizes the need for standardized training and evaluation procedures to improve accuracy.	Standardization in model training and evaluation enhances diagnostic performance and reliability of deep learning models in pancreatic cancer detection.

Overall, this table encapsulates the essential aspects of standardization as discussed in these studies, providing a clear perspective on how consistent methodologies contribute to advancements in diagnostic technology and practice.

### 4.4. Advancing Diagnostic Techniques: The Intersection of AI with Cytopathology, Histopathology, and Radiology

#### 4.4.1. AI Trends in Cytopathology Compared with Histopathology and Radiology

It is insightful to examine the findings of this review in the context of other imaging domains, particularly digital pathology, which intersects with cytology and cytopathology. A comparative analysis with radiology is especially pertinent, given radiology’s accelerated integration into digital health, primarily because of its early adoption of DICOM standards.

Radiology’s swift digital adoption offers valuable lessons for digital pathology. The rapid and widespread implementation of standardized protocols in radiology has significantly facilitated the integration of advanced technologies, such as AI, into clinical practice. Emulating these standardization and interoperability measures could expedite similar advancements in digital pathology. Radiology’s experience underscores the crucial role of well-defined standards in enhancing technology integration and optimizing clinical workflows. By adopting analogous strategies, digital pathology could experience substantial progress and smoother incorporation of new technologies.

In contrast, the scientific output in histopathology, which began in 1988—ten years earlier than cytopathology’s start in 1998 [56,57]—reflects a more extensive body of research. For instance, there are (at the date of this review of 15 July 2024) 1616 publications related to histopathology and AI compared with just 101 in cytopathology and AI [56] based on a PubMed search (see keys in Box 2). This disparity likely arises from the greater challenges in managing digital cytology and cytopathology, where sophisticated solutions and significant memory resources are required due to the complexity of focus functions [58].

PubMed data further highlights this trend: there are 335 review articles in histopathology, including 36 systematic reviews, reflecting a proportion consistent with its larger research volume [57]. In the past five years, there has been a notable acceleration in AI-related publications in histopathology (Figure 8 and Figure 9).

Historically, AI-related research in histopathology constitutes about 1.84% of its total publications, a figure that has risen to 4.84% in the last five years, aligning closely with cytopathology’s rate (Figure 10 and Figure 11).

In comparison, radiology’s scientific output, which began 15 years earlier in 1983 [59], is significantly more extensive. Radiology has 3078 publications related to AI (Figure 12), compared with just 101 in cytopathology and AI [56,59] based on a PubMed search (see Box 3 for the used keys), This difference is attributable to the earlier and more rapid standardization and digital integration in radiology [58].

**Figure 15:**
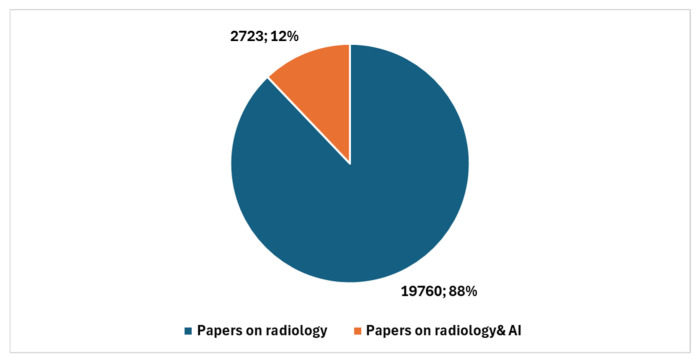
Ratio of AI-Focused to Non-AI-Focused Research in radiology in the last five years.

PubMed data shows 830 review articles in radiology, including 95 systematic reviews, maintaining a proportion that reflects its larger body of research [59] (Figure 13). Recent trends indicate a substantial increase in AI-related publications in radiology over the past five years (Figure 14 and Figure 15).

Historically, AI-related research in radiology represents 4.41% of the total publications, significantly higher than the sub-2% figures seen in both histopathology and cytopathology. In the past five years, this proportion has surged to 12.11%, nearly 2.5 times higher than the rates observed in cytopathology and histopathology, where it was below 5%. (Figure 14 and Figure 15).

The rapid advancement and integration of AI in radiology provide a compelling model for other imaging domains, including cytopathology and histopathology. Radiology’s success in adopting standardized protocols and embracing digital technologies serves as a valuable blueprint for accelerating similar progress in these fields. By drawing inspiration from radiology’s approach to digital health, both cytopathology and histopathology can leverage these insights to overcome existing challenges, streamline workflows, and enhance the integration of AI technologies.

In conclusion, the table reported in the following and the figures presented before provide a comprehensive overview of scientific production in histopathology and radiology, focusing on the impact of artificial intelligence (AI). The table offers a comparative analysis of the total number of publications, review articles, and the proportion of AI-related research in both fields. This comparison highlights the significant differences in research volume and focus between histopathology and radiology. The figures below, anticipated in the text, illustrate key trends and ratios:

Figure 8 and Figure 9 show the temporal trends and distribution of review and systematic review articles in histopathology.

Figure 10 and Figure 11 present the ratio of AI-focused to non-AI-focused research in histopathology, including trends over the last five years.

Figure 12 and Figure 13 detail the temporal trends and review article distribution in radiology&AI.

Figure 14 and Figure 15 highlight the ratio of AI-focused research in radiology, emphasizing recent trends and allowing comparisons with histopathology.

These visualizations underscore the evolving focus on AI across these fields, revealing the rapid advancements and integration of AI technologies, particularly in radiology. Comparative data provide valuable insights into how AI is shaping research and clinical practices in medical imaging.

Table 10 reports a sketch of the Distribution and Trends of AI-Focused Scientific Publications in Histopathology, Cytopathology, and Radiology.

Box 3 **Keys used for the Pubmed searches.**

*(Cytopathology [Title/Abstract]) AND ((Artificial intelligence [Title/Abstract]) OR (machine learning [Title/Abstract]) OR (deep learning [Title/Abstract]) OR (neural network [Title/Abstract]))*

*(histopathology [Title/Abstract]) AND ((Artificial intelligence [Title/Abstract]) OR (machine learning [Title/Abstract]) OR (deep learning [Title/Abstract]) OR (neural network [Title/Abstract]))*

*(radiology [Title/Abstract]) AND ((Artificial intelligence [Title/Abstract]) OR (machine learning [Title/Abstract]) OR (deep learning [Title/Abstract]) OR (neural network [Title/Abstract]))*

*(histopathology [Title/Abstract])*

*(Cytopathology [Title/Abstract])*

*(radiology [Title/Abstract])*

#### 4.4.2. Strategic Directions of Advancements in Cytopathology: Lessons from Histopathology and Radiology Comparisons

The integration of artificial intelligence (AI) into cytopathology is significantly hindered by challenges related to the specificity of the digitization process. These challenges render the integration complex and costly, particularly when compared with the more advanced integration seen in histopathology and digital radiology.

Cytopathology, similar to histopathology, is part of the broader digital pathology field, yet it lags in the incorporation of AI technologies. Histopathology has a more established pathway due to its shared focus on digital pathology, allowing it to navigate the integration process more smoothly. In contrast, digital radiology has experienced a more rapid evolution, achieving substantial milestones and advancements ahead of both cytopathology and histopathology.

This umbrella review aims to address not only the current challenges and trade-offs encountered in the integration of AI within cytopathology as emerged from the overview but also to explore valuable lessons and recommendations. By drawing insights from histopathology—an area with which cytopathology shares the digital pathology framework—and from digital radiology, which has progressed more swiftly, this review seeks to highlight effective strategies and potential solutions. These insights will be crucial for overcoming the barriers faced by cytopathology and aligning its progress with that of its counterparts in the digital health ecosystem.

##### Expanding Diagnostic Horizons: Insights from Histopathology for AI Integration in Cytopathology

As digital health evolves, integrating artificial intelligence (AI) into cytopathology poses unique challenges compared with advancements in histopathology. Traditional histopathology has faced barriers such as low throughput and interobserver variability, hampering progress in precision medicine. However, innovations in computational pathology, particularly through deep learning (DL) techniques, are overcoming these barriers [60,61,62].

Advancements in Histopathology and Computational Pathology

- DL models have significantly improved diagnostic tasks in histopathology, excelling in mutation prediction, large-scale pathomics analyses, and prognosis forecasting.
- The integration of multimodal data and the development of foundation models in computational pathology opens new diagnostic possibilities [60].
- **Implication for Cytopathology**: Similar advancements can enhance cytopathology, but a customized AI integration approach is essential to address its unique challenges.

The Role of Digital Pathology and AI Integration

- Digital pathology (DP) transforms clinical practice by converting glass slides into high-resolution whole-slide images (WSI), improving quality assurance and diagnostic accuracy.
- AI applications in DP, such as Immunoscore (IS) and Immunoscore-Immune Checkpoint (IS-IC), have shown great promise in cancer diagnostics [61].
- **Implication for Cytopathology**: AI tools similar to these can refine diagnostic processes in cytopathology, enhancing both precision and efficiency.

Challenges in AI Adoption and Utilization

- A study among Polish pathologists revealed a knowledge gap in AI applications, highlighting the need for improved awareness and education [62].
- **Implication for Cytopathology**: Targeted educational initiatives are crucial to promote AI adoption in cytopathology.

##### Lessons from Radiology for AI Integration in Cytopathology

AI is rapidly transforming radiology, offering valuable insights for cytopathology. Key lessons include [63,64,65,66,67,68]:

Enhancing Diagnostic Accuracy and Workflow

- AI has improved radiology’s diagnostic accuracy and efficiency by automating tasks and aiding in detecting abnormalities [63,64].
- **Lesson for Cytopathology**: AI-driven image analysis can enhance the detection of typical cellular patterns, reducing false negatives and improving diagnostic precision.

Addressing Legal and Ethical Challenges

- The integration of AI into radiology presents legal and ethical issues, including liability and transparency concerns [63].
- **Lesson for Cytopathology**: Develop clear liability guidelines and transparent AI systems to facilitate responsible AI use in diagnostics.

Bridging Knowledge Gaps and Training Needs

- Radiologists face significant knowledge gaps in AI applications, making education and training crucial [67].
- **Lesson for Cytopathology**: Invest in comprehensive AI training programs to ensure effective AI integration and maximize diagnostic outcomes.

Understanding Cognitive and Contextual Challenges

- AI decision-making models differ from human processes, complicating clinical practice integration [65].
- **Lesson for Cytopathology**: Ensure AI tools complement human expertise by aligning them with the unique decision-making processes of cytopathology.

Leveraging Advanced Technologies

- Technologies such as natural image captioning (NIC) have enhanced radiology report generation and documentation [66].
- **Lesson for Cytopathology**: Adapt similar technologies for cytopathology to streamline report generation and improve communication.

##### Suggestions for Advancing AI Integration in Cytopathology: Lessons from Histopathology and Radiology

Drawing from histopathology and radiology, several key strategies can be applied to cytopathology for enhanced AI adoption:

1. Conduct Comprehensive Acceptance and Utilization Studies
  - Studies in histopathology and radiology underscore the need for evaluating knowledge, attitudes, and practices regarding AI [62,67].
    ○ **Action**: Implement questionnaires to gauge cytopathologists’ familiarity and attitudes towards AI. Identify gaps in education and training.
    ○ **Action**: Evaluate barriers to AI adoption in workflow, data management, and training to design effective strategies.
2. Foster Cross-Disciplinary Collaboration and Training
  - Collaboration between histopathology, radiology, and cytopathology drives AI tool development [63,64].
    ○ **Action:** Create interdisciplinary training programs and promote collaborative research across fields.
    ○ **Benefit:** Joint efforts can lead to integrated AI tools that enhance diagnostic accuracy and efficiency.
3. Explore Multidimensional Data Integration
  - Combining radiomic and pathognomic data has expanded disease understanding in radiology [68].
    ○ **Action:** Develop AI models integrating cytopathological data with radiographic and histopathological information.
    ○ **Benefit:** This multimodal approach can improve diagnostic precision and prognostic assessments.
4. Address Legal, Ethical, and Transparency Issues
  - Legal and ethical concerns in radiology highlight the need for clear guidelines and transparency in AI systems [63].
    ○ **Action:** Establish comprehensive legal and ethical guidelines for AI use in cytopathology.
    ○ **Action:** Ensure AI systems are transparent and explainable to build trust and facilitate clinical integration.
5. Enhance Reporting and Documentation
  - Radiology has improved report generation with advanced AI-driven tools [66].
    ○ **Action:** Implement AI-driven reporting tools to improve the clarity, consistency, and completeness of cytopathological reports.
    ○ **Action:** Utilize natural language processing (NLP) to automate report generation, ensuring comprehensive clinical decision-making support.
6. Expand Knowledge and Attitude Surveys
  - Expanding surveys can help identify gaps in knowledge and attitudes toward AI across medical fields [67].
    ○ **Action:** Develop targeted questionnaires to assess cytopathologists’ understanding of AI and their concerns regarding its integration.
    ○ **Action:** Use survey data to design tailored educational programs and resources for cytopathologists.

By implementing these suggestions, cytopathology can effectively advance its AI integration efforts, drawing on lessons from histopathology and radiology. Comprehensive acceptance studies, interdisciplinary collaboration, and multimodal data integration will be critical for optimizing the potential of AI in cytopathology.

##### From Histopathology and Radiology to Cytopathology: Final Scheme for Enhancing AI Integration

To facilitate a comprehensive understanding of how advancements in histopathology and radiology can inform the integration of artificial intelligence (AI) in cytopathology, we present a detailed overview of the key lessons and strategies derived from these fields. The following Table 11 summarizes critical insights and recommendations based on recent studies and reviews. These insights highlight both the advancements achieved and the challenges faced in histopathology and radiology, which can be adapted to enhance AI integration in cytopathology.

### 4.5. Limitations

The proposed study, described as a narrative review, has inherent limitations due to the methodology and inclusion/exclusion criteria applied. The absence of conference studies results in a potential lack of updates on ongoing research and recent advances, excluding preliminary data that may not have been officially published yet. Additionally, the exclusion of local studies and/or guidelines in local languages restricts the analysis to contributions published in English and international guidelines, potentially overlooking specific and relevant insights from particular cultural or clinical contexts. This limits the understanding of regional variations in clinical practices and treatment protocols.

## 5. Final Reflections: Broadening Ethical Considerations in AI Applications Cytopathology

The study highlighted that one of the key strategic aspects for the future of AI in cytopathology is the need to address ethical concerns coherently and appropriately. The umbrella review provided evidence from review articles, but complementary analyses of primary studies revealed that scholars have not yet conducted targeted research specifically focused on ethics in this field. However, it is clear that three emerging ethical polarities in AI within healthcare can be directly applied to cytopathology.

### 5.1. Emerging Ethical Polarities in AI and Their Implications for Cytopathology

#### 5.1.1. Expansion of Algorethics in Healthcare

The first polarity concerns the growth of “algorethics”—the ethics of algorithms—which has expanded beyond the biomedical literature into national and international policy frameworks [69]. Organizations such as the World Health Organization (WHO), the European Union (EU), and various national regulatory bodies are establishing ethical standards that can influence AI applications, including in cytopathology.

In cytopathology, the application of AI-powered diagnostic tools, such as algorithms for automating the detection of abnormal cells, raises ethical issues regarding the transparency of algorithmic decisions and the accountability of automated systems. Ethical frameworks from WHO, the EU AI Act, and the U.S. FDA offer essential guidelines that can be adapted to ensure these technologies are developed in ways that safeguard patient rights, maintain accuracy, and promote equity in cytological diagnostics. In detail, the integration of AI into cytopathology brings profound ethical implications, which are increasingly being addressed through various global and national frameworks. These frameworks, although not always specifically focused on cytopathology, significantly impact the field by shaping ethical practices and standards for AI technologies. The following key areas illustrate this impact:

Ethical Standards and Equity: The WHO global AI ethics guidelines stress the importance of respecting human rights and promoting health equity [70]. These guidelines aim to ensure that AI applications in cytopathology are developed and utilized in ways that contribute positively to health outcomes while maintaining ethical standards.

Regulatory Frameworks: The EU AI Act represents a comprehensive effort to regulate AI usage across member states, focusing on ethical practices, transparency, and accountability [71]. This regulatory framework addresses how AI technologies should be implemented in cytopathology, striving for a balance between innovation and ethical responsibility.

Transparency and Safety: FDA guidelines on AI in medical research emphasize the need for transparency and safeguarding public health [72,73]. These guidelines are critical for ensuring that AI technologies in cytopathology are effective and ethically deployed, fostering trust in AI applications and protecting patient welfare.

Risk Management and Ethical Assurance: The NHS AI Ethics Initiative supports the ethical integration of AI within healthcare settings, including cytopathology, by providing assurance and managing associated risks [74]. This initiative focuses on maintaining high ethical standards in diagnostic practices, ensuring that AI systems are used responsibly and effectively.

Responsible Practices and Data Protection: The Public Health Agency of Canada has developed an ethical framework for AI applications in public health, which emphasizes responsible practices and personal data protection [75]. This framework guides the ethical deployment of AI technologies in cytopathology, ensuring that patient data privacy is safeguarded while enhancing diagnostic capabilities.

Ethical Norms and Enforcement: The CSET reports ethical norms for AI use in China, which cover the use and protection of personal information and human control over AI [76]. These norms can also influence the ethical deployment of AI in cytopathology, ensuring responsible practices are upheld.

Overall, while these documents are not exclusively focused on cytopathology, they profoundly influence the field by establishing ethical principles and standards for AI technologies. Ensuring that AI is developed and implemented in responsible, equitable, and ethically aligned ways is essential for addressing the complex challenges posed by AI in cytopathology.

#### 5.1.2. Impact of Chatbots and Large Language Models (LLMs)

The second polarity involves the transformative impact of chatbots and large language models (LLMs) [27], which are already revolutionizing areas such as radiology and are poised to achieve the same in cytopathology. These technologies can automate diagnostic reporting, enhance communication, and support cytopathologists by providing real-time assistance in interpreting results.

In cytopathology, LLMs could assist with the initial screening of cytological samples or automate the communication of findings to clinicians. However, their implementation introduces ethical challenges, particularly around decision-making transparency, the potential for over-reliance on automated systems, and ensuring that patient data are handled responsibly. Clear ethical guidelines are necessary to ensure that AI-enhanced cytopathological diagnostics remain accurate and accountable while preserving trust in the healthcare system.

#### 5.1.3. Role of Telemedicine and AI in Cytopathology

The third polarity is the increasing integration of telemedicine into various healthcare disciplines, including cytopathology [77]. Telemedicine is expanding access to diagnostic services by enabling remote analysis and consultations, but it also presents ethical issues, particularly regarding equitable access, the accuracy of remote diagnostics, and the secure transmission of patient data.

In cytopathology, the digital transmission of cytological images and patient data for remote analysis is becoming more common. While telemedicine can enhance diagnostic efficiency and accessibility, the integration of AI tools in this context requires stringent ethical oversight. Ensuring data privacy, maintaining diagnostic accuracy, and addressing disparities in access are key ethical concerns that must be managed as telemedicine becomes a standard practice in cytopathological workflows.

## 6. Conclusions

This umbrella review highlights the transformative potential of artificial intelligence (AI) in cytopathology, emphasizing the need to address a range of challenges for successful integration. The integration of AI technologies is poised to enhance diagnostic accuracy and operational efficiency within cytopathology significantly. By automating and refining diagnostic processes, AI has the potential to reduce errors and improve patient outcomes. The ongoing evolution of AI applications presents exciting opportunities to revolutionize diagnostic practices and patient management.

However, this review also identifies several critical challenges that must be overcome to leverage the benefits of AI in cytopathology fully. Key issues include:

Data Standardization: A significant barrier to AI integration is the lack of standardized data formats and terminology within cytopathology. Ensuring consistency in data collection and representation is essential for developing effective AI algorithms that can generalize across diverse datasets.

Integration with Existing Clinical Workflows: The successful implementation of AI tools requires seamless integration with existing clinical workflows. This necessitates collaboration between IT specialists, cytopathologists, and other healthcare professionals to ensure that AI applications fit naturally into routine practices without disrupting patient care.

Ethical and Legal Concerns: Addressing ethical dilemmas related to patient privacy, data security, and algorithmic biases is crucial. Establishing clear guidelines and governance frameworks will help navigate these complexities and ensure responsible AI deployment in clinical settings.

Validation and Quality Assurance: Rigorous validation of AI tools is essential to establish their reliability and accuracy. Continuous monitoring and quality assurance mechanisms should be implemented to evaluate AI performance over time, ensuring that these tools consistently meet clinical standards.

Key Insights:

Opportunities: AI demonstrates significant potential in enhancing diagnostic accuracy, improving workflow efficiency, and offering novel insights into cytopathology. Successful implementations in related fields such as histopathology and radiology provide valuable models for accelerating AI adoption in cytopathology.

Challenges: Major hurdles include the need for rigorous validation, data standardization, and addressing biases. Additionally, high costs and data quality issues must be managed to realize the benefits of AI fully.

Future Directions: To advance AI in cytopathology, it is crucial to apply lessons learned from histopathology and radiology while fostering focused research to tackle existing barriers. Addressing challenges related to data standardization, integration, and ethical considerations will be pivotal for effective AI integration, ultimately leading to improved diagnostic practices in cytopathology.

## Figures and Tables

**Table 1:** Research areas/focus and queries.

Theme	Research Focus	Query
Deep Learning Algorithms and Cell Image Analysis	Development of AI models for detailed cellular structure analysis	“Deep Learning Algorithms AND Cell Image Analysis in Cytopathology”
		
Digital Imaging & Digital Pathology Tools	Use of digital platforms and images for AI-assisted cytological analysis	“Digital Imaging OR Digital Pathology Tools for AI-based Cytology”
		
AI-assisted Diagnosis & Predictive Analytics	AI supporting diagnostic decisions and predicting clinical outcomes	“AI-assisted Diagnosis AND Predictive Analytics in Cytopathology”
		
Automated Diagnostic Systems & Computer-Aided Diagnosis	Full or partial automation of cytology diagnostics using AI	“Automated Diagnostic Systems OR Computer-Aided Diagnosis with AI”
		
Algorithmic Classification of Cells & Image Recognition	Use of AI algorithms to classify cells based on image recognition	“Algorithmic Classification of Cells AND Image Recognition in Cytopathology”
		
Machine Learning & AI-based Cytological Screening	Application of ML algorithms for cytological screening processes	“Machine Learning AND AI-based Cytological Screening”
		
AI-driven Cytopathological Innovation & Diagnostic Precision	Innovations and precision improvements in cytopathology through AI	“AI-driven Cytopathological Innovation OR Diagnostic Precision with AI”
		
AI in Medical Imaging & Cytopathological Workflow Optimization	AI applied to medical imaging and optimizing the cytopathology diagnostic workflow	“AI in Medical Imaging AND Cytopathological Workflow Optimization”
		
Remote Cytopathology Consultations & AI-assisted Diagnosis	Use of AI for remote diagnosis and consultations in cytopathology	“Remote Cytopathology Consultations AND AI-assisted Diagnosis”
		
AI and Pathologist Collaboration & AI-based Cytological Screening	Research on the collaboration between AI systems and pathologists for screening and diagnosis	“AI and Pathologist Collaboration OR AI-based Cytological Screening”
		
General Search	Broad and focused research on AI, machine learning, deep learning, and neural networks in cytopathology	“(Cytopathology [Title/Abstract]) AND ((Artificial intelligence [Title/Abstract]) OR (machine learning [Title/Abstract]) OR (deep learning [Title/Abstract]) OR (neural network [Title/Abstract]))”

**Table 10:** Distribution and Trends of AI-Focused Scientific Publications in Histopathology, Cytopathology, and Radiology.

	Start Year	Total Publications	AI Publications	Percentage of AI Publications	Review Articles	Systematic Reviews Among the Review Articles	AI Research Ratio (Last 5 Years)
**Histopathology**	1988	88,585	1.616	1.84%	335	36	4.84%
**Cytopathology**	1998	5682	101	1.79%	31	1	4.95%
**Radiology**	1983	69,725	3.078	4.84%	830	95	12.11%

**Table 11:** Emerging recommendations from histopathology and radiology domain for AI Integration in cytopathology.

Aspect	Histopathology Insights	Radiology Insights	Recommendations for Cytopathology	References
Acceptance and Utilization	- Evaluating knowledge, attitudes, and practices is crucial [62].	- Surveys show variable adoption and training needs [67].	- Conduct detailed surveys to assess cytopathologists’ knowledge and attitudes towards AI. Address barriers to adoption.	[60,61,62,67]
				
Cross-Disciplinary Collaboration	- Collaboration enhances the development and implementation of AI tools [60].	- Interdisciplinary knowledge sharing improves AI integration [64].	- Develop joint training programs involving histopathologists, radiologists, and cytopathologists. Foster interdisciplinary research.	[60,64]
				
Multidimensional Data Integration	- Integration of multimodal data is advancing diagnostic capabilities [60].	- Combining radiomic and pathognomic features enhances diagnostic accuracy [68].	- Develop AI models that integrate cytopathological data with other diagnostic modalities. Explore advanced methodologies for data integration.	[60,68]
				
Legal and Ethical Issues	- AI use raises legal responsibility and transparency concerns [63].	- Legal responsibility and ethical considerations are complex [63].	- Establish clear guidelines for AI use in cytopathology, including liability, data privacy, and transparency.	[63]
				
Reporting and Documentation	- Advanced reporting tools enhance clarity and completeness [61].	- AI-driven reporting improves documentation quality [66].	- Implement AI-driven reporting tools to enhance the clarity and completeness of cytopathological reports. Utilize NLP for automation.	[61,66]
				
*Knowledge and Attitude Surveys*	- Increased awareness and education are needed for effective AI integration [62].	- Understanding knowledge gaps and attitudes is essential [67].	- Design and deploy targeted questionnaires to assess and improve cytopathologists’ understanding and use of AI.	[62,67]

## Supplementary Materials

The following supporting information can be downloaded at: https://www.mdpi.com/article/10.3390/jcm13226745/s1, Section S1: Analytical Summaries for reviews [21,22,23,24,25,26,27,28,29,30,31,32,33,34,35,36,37,38,39,40,41] in Section 3; Section S2: Analytical summaries for *primary studies* [42,43,44,45,46,47,48,49,50,51,52,53,54,55] in Section 4.
