# Supplementary material for: AI in Cytopathology: A Narrative Umbrella Review on Innovations, Challenges, and Future Directions
Source: J Clin Med. 2024 Nov 9;13(22):6745. doi: 10.3390/jcm13226745 (PMC11594881; doi:10.3390/jcm13226745)
Supplement: Supplementary file 1 [file jcm-13-06745-s001.zip › jcm-3184833-supplementary.pdf]

Journal of
Clinical Medicine

MDPI

Review

AI in Cytopathology: a narrative umbrella review on Innovations, Challenges, and Future Directions

Daniele Giansanti *

Centro TISP, ISS via Regina Elena 299,00161,Rome, Italy
* Correspondence: daniele.giansanti@iss.it

s.1 Analytical Summaries for reviews [21-41] in section 3

The overview has been complemented by means of analytical summaries (AS)swith focus on the contribution of the *AI in cytopathology*, in the following the Ass of the singles reviews (in italics is reported the theme).

Computer-Assisted Diagnosis (CAD) in Urine Cytopathology

Ciaparrone et al. (2024) [21] delve into the role of CAD systems in urine cytopathology, focusing on their potential to enhance the diagnosis of urothelial carcinomas. This review underscores the transformative impact of CAD technologies, which utilize machine learning algorithms to analyze cytology samples more accurately and efficiently than traditional methods. CAD tools can improve diagnostic accuracy by identifying subtle patterns and anomalies that may be missed by human reviewers. The review highlights several CAD models and their methodologies, emphasizing their ability to streamline workflows, reduce diagnostic errors, and uncover novel biomarkers. However, the integration of CAD into routine clinical practice poses challenges, including the need for rigorous validation, regulatory approval, and comprehensive training for pathologists. The study advocates for continued research to address these challenges and fully harness CAD's potential in improving patient care.

AI-Assisted Diagnosis of Thyroid Cancer

Zhang et al. (2024) [22] explore how AI technologies are revolutionizing the early diagnosis of thyroid cancer (TC). Thyroid cancer is increasingly common, and early detection is crucial for effective treatment. The review highlights AI's application in analysing ultrasound images, cytopathology samples, and molecular markers to assess the risk of malignancy in thyroid nodules. AI models have demonstrated performance comparable to experienced clinicians, suggesting that they could significantly enhance early diagnosis and risk stratification. The study emphasizes the potential of AI to improve diagnostic accuracy and efficiency, although it also notes that further development and clinical validation are necessary to refine these tools and expand their use in routine practice.

The Evolving Role of Cytopathology in the Molecular and Computational Era

Caputo et al. (2024) [23] provide an overview of how cytopathology is adapting to the increasing complexity of diagnostic practices through molecular and computational advancements. The paper discusses how next-generation sequencing and digital pathology are enhancing cytopathology's ability to diagnose and stratify cancer risk more precisely. AI tools are seen as complementary to molecular investigations, offering additional layers of diagnostic insight and improving the cost-effectiveness of large-scale screening programs. The study highlights the benefits of digital and AI technologies in handling complex and intermediate cytology cases, suggesting that these advancements could significantly enhance diagnostic accuracy and efficiency. It also offers a perspective on how young pathologists view these evolving tools and their integration into practice.

Integrated Diagnostics for Thyroid Nodules

Citation: To be added by editorial staff during production.

Academic Editor: Firstname Lastname

Received: date
Revised: date
Accepted: date
Published: date

Copyright: © 2024 by the authors. Submitted for possible open access publication under the terms and conditions of the Creative Commons Attribution (CC BY) license (https://creativecommons.org/licenses/by/4.0/).

Giovanella et al. (2024) [24] discuss the integration of various diagnostic tools for evaluating thyroid nodules, emphasizing a multimodal approach. The paper reviews the use of ultrasound, fine-needle aspiration cytology (FNAC), molecular imaging, and AI in diagnosing thyroid nodules. Each method's strengths and limitations are analysed, with a focus on how these tools can be effectively combined to improve diagnostic accuracy and minimize unnecessary procedures. The study suggests that while ultrasound scoring systems and FNAC are valuable, AI and molecular imaging can further refine diagnosis and reduce the need for "diagnostic surgery." The clinical value of AI in this context is highlighted, though the authors call for additional research to fully establish its role and effectiveness.

Digital Cytology and AI Integration

Kim et al. (2024) [25] present in a first part of a study a comprehensive review of digital cytology and AI integration in cytopathology, prepared by a task force from the American Society of Cytopathology. The review addresses the growing adoption of digital cytology, including whole-slide imaging, and the integration of AI technologies into cytology workflows. The paper examines the potential benefits of these technologies, such as improved diagnostic accuracy, efficiency, and workflow management. It also highlights the current state of AI implementation in laboratories, based on a global survey and virtual roundtable discussions. The review provides best practice recommendations for incorporating digital cytology and AI into routine practice and discusses legal considerations related to their use. This study aims to guide laboratories in effectively adopting and utilizing these advanced technologies.

Kim et al. (2024) in the second part of a study [26] focus on the practical implementation of digital cytology within laboratory settings. This first part of a two-part white paper from the American Society of Cytopathology provides a comprehensive review of how digital cytology, including whole-slide imaging and AI applications, can be integrated into cytology practice. The task force evaluated current literature, conducted a global survey, and engaged in discussions with industry representatives. The paper outlines best practice recommendations for adopting digital cytology, including considerations for workflow integration, training, and infrastructure. It highlights the potential benefits of digital cytology, such as improved diagnostic efficiency and accessibility, while also addressing challenges like technology costs and the need for standardized protocols. The study serves as a guideline for laboratories looking to incorporate these advanced technologies into their routine practices.

ChatGPT in Pathological Diagnosis of Cancer

Malik and Zaheer (2024) [27] explore the role of ChatGPT, an AI-based chatbot developed by OpenAI, in aiding cancer diagnosis. The paper discusses how integrating AI and digital slides into pathology workflows can enhance diagnostic processes by providing additional layers of analysis and knowledge synthesis. ChatGPT can assist pathologists by processing large volumes of data and offering insights that go beyond traditional microscopic evaluation. Despite its potential, the study acknowledges challenges such as integrating AI with existing systems, addressing biases, and navigating legal issues. The paper calls for further development and research to address these challenges and fully realize AI's potential in improving cancer diagnosis.

Machine and Deep Learning in Thyroid Cytology and Histopathology

Slabaugh et al. (2023) [28] review the application of machine learning (ML) and deep learning (DL) technologies in thyroid cytology and histopathology. The review emphasizes how these technologies can address limitations in current diagnostic practices, such as high interobserver variability and indeterminate fine-needle aspiration cytology (FNAC) results. ML and DL algorithms have shown promise in classifying thyroid lesions, particularly papillary thyroid carcinoma. However, many studies are retrospective, and there is a need for prospective validation and practical implementation in clinical settings. The paper highlights the need for further research into improving algorithm interpretability, multiclassification capabilities, and integration into diverse clinical workflows.

Insights from the 2022 WHO Classification of Thyroid Neoplasms

Lebrun and Salmon (2024) [29] review updates from the 2022 WHO classification of thyroid neoplasms and the 2023 Bethesda System for Reporting Thyroid Cytopathology (TBSRTC). The review discusses how these updates, including new entities and the integration of molecular testing, can refine the diagnosis and risk stratification of thyroid cancer. FNAC remains a key diagnostic tool, but molecular testing enhances its utility by improving risk assessment. The paper outlines the importance of integrating morphological, molecular, and clinical data to achieve accurate diagnosis and prognosis. It also notes ongoing challenges, such as the management of low-risk neoplasms and oncocytic lesions, and suggests that AI could play a crucial role in future diagnostic and therapeutic strategies.

AI in Fungal Cytopathology

Singla et al. (2024) [30] review the application of AI in fungal cytology, focusing on its potential to transform the detection and typing of fungal infections. AI technologies, including machine learning algorithms, have advanced the identification of fungi by analyzing complex data sets and providing real-time identification tools. The review highlights AI's success in various areas of cytopathology, including thyroid, breast, urine, and cervical lesions, and anticipates similar benefits for fungal cytology. The paper discusses AI's ability to handle large volumes of data with accuracy and reproducibility and emphasizes the need for further research to explore its full potential in fungal cytology. The review also provides a brief overview of AI basics and its implications for future diagnostic applications.

Biomarker Integration in Oral Cytopathology

Sunny et al. (2023) [31] propose a study addressing the challenge of diagnosing oral potentially-malignant disorders, which are prevalent and carry varying risks of malignant transformation. Traditional oral cytology has limitations due to subjective interpretation and a lack of definitive diagnostic criteria. The study identifies a panel of biomarkers (CD44, Cyclin D1, SNA-1, and MAA) and evaluates their effectiveness in distinguishing high-grade dysplastic lesions from benign or low-risk lesions. By integrating these biomarkers with machine learning, the study improves diagnostic sensitivity and specificity. The findings suggest that combining a two-biomarker panel with automated image analysis could enhance risk stratification and facilitate point-of-care diagnostics, potentially reducing the need for invasive procedures and improving patient management.

Machine Learning in Thyroid Cytopathology

Wong et al' review (2023)[32] explores the integration of machine learning (ML) with digital pathology, specifically focusing on thyroid cytopathology. While digital pathology has made strides in analyzing tissue specimens, cytology specimens present unique challenges due to scanning difficulties and the need for optimized imaging technology. Despite these challenges, ML algorithms have shown promise in thyroid fine needle aspiration biopsy (FNAB) specimens, improving diagnostic accuracy and workflow efficiency. The review highlights the potential of ML to enhance thyroid cancer diagnosis but also stresses the need for larger, diverse datasets and further validation studies to refine these algorithms and integrate them into clinical practice effectively.

AI in Thyroid Nodule Diagnosis and Classification

This Ludwig et al.' (2023) [33] review focuses on the role of artificial intelligence (AI) in diagnosing and classifying thyroid nodules, a common clinical issue given the rising incidence of thyroid nodules. AI's applications in ultrasonography and pathology for thyroid disease diagnosis are discussed. The review synthesizes recent innovations and identifies AI's potential to improve the classification and management of thyroid nodules, potentially reducing unnecessary procedures and improving diagnostic accuracy. The study underscores the importance of ongoing research to validate AI tools, enhance their clinical applicability, and ensure they meet practical needs in thyroid disease management.

AI in Microbiological Disease Diagnosis

Marletta et al. (2023) [34] propose a study with the focus on the application of AI in diagnosing microbiological diseases, particularly in resource-limited settings. AI's role in identifying various microorganisms, including malaria, bacteria, and nematodes, is evaluated through a systematic search of literature. The study highlights AI's potential to assist pathologists by analyzing cytological images and improving diagnostic accuracy. The review suggests that AI could play a critical role in enhancing microbiological diagnostics, especially in settings where manual examination is challenging. Technological improvements and better datasets are necessary to expand AI's adoption and effectiveness in these contexts.

AI in Thyroid Nodule Evaluation

Tessler et al. (2022) [35] remarks that AIhas increasingly been applied to the evaluation of thyroid nodules, leveraging both machine learning and deep learning techniques. Machine learning involves algorithms that learn from data patterns provided by humans, while deep learning uses neural networks to mimic brain functions for data analysis. AI's role in thyroid disease has been primarily in the risk stratification of malignancy in nodules, with several platforms receiving FDA approval. These AI systems aim to enhance diagnostic accuracy and efficiency, particularly benefiting physicians with less experience. While validation studies have shown mixed results, there is promising evidence that AI can outperform some human diagnosticians. Ongoing challenges include ensuring the practical usability and cost-effectiveness of AI tools in real-world clinical settings.

AI in Cytopathology of Non-Gynaecological Cancers

The systematic review by Thakur et al. (2022) [37] explores AI applications in non-gynaecological cancer cytopathology, focusing on various cancer types, including thyroid, bladder, lung, and pancreas. The review evaluates different AI models used for image classification and segmentation tasks, noting that while results have been promising, challenges remain in dataset size and quality. The study emphasizes the need for larger, well-annotated datasets and external validation to improve AI models and their application in clinical settings. The review concludes that AI holds significant potential for enhancing non-gynecological cancer diagnostics but requires further development and validation.

Overview of AI in Cytopathology

This review by Alrafiah et al. (2022) [38] provides a comprehensive overview of AI advancements in cytopathology, from its initial use in Pap test screening to current applications in analysing both gynaecological and non-gynaecological cytology specimens. The review highlights the evolution of AI technologies, including deep learning and machine learning, and their impact on diagnostic accuracy. It discusses the need for transparency in AI systems, robust validation studies, and practical integration into clinical workflows. The review concludes that AI has the potential to significantly improve cytopathology diagnostics but requires ongoing research, development, and validation to ensure its effectiveness and applicability in real-world settings.

Artificial Intelligence Applications in Cytopathology state of art

The review by Vaikus et al [39] discusses recent advancements in cytopathology through the development of consensus rule sets (e.g., Bethesda, Milan, Paris), which aim to enhance diagnostic consistency and clarity. These systems work by reducing diagnostic variability, eliminating ambiguous categories, and promoting quantitative scoring. The paper highlights how computational pathology, which leverages artificial intelligence, is a natural extension of these advancements. AI offers the potential for fully reproducible and objective diagnoses, addressing biases and variability inherent in human interpretation. This integration of AI into cytopathology is expected to further refine diagnostic processes and improve accuracy.

Urinary tract cytopathology and AI

Jorda et al . [40] recalls that urine cytology is a non-invasive, cost-efficient, and sensitive test for detecting high-grade urothelial carcinoma. The Paris System (TPS) for Reporting Urinary Cytology, an evidence-based framework, employs malignancy risk to guide patient management. Since its introduction, TPS has standardized urine cytology

reports, enhancing communication between pathologists and clinicians. However, to minimize the risk of false-negative and false-positive results, it is crucial to correlate urine cytology findings with concurrent tissue samples whenever possible. To address these challenges, several ancillary tests and advanced artificial intelligence (AI) algorithms are being developed. AI technologies are being integrated into urine cytology to further enhance diagnostic accuracy. These AI-driven tools can analyze cytological images with high precision, identify subtle patterns that might be missed by human observers, and provide consistent and reproducible results. By leveraging large datasets and sophisticated algorithms, AI can help improve the sensitivity and specificity of urine cytology, ultimately leading to better patient outcomes and more effective management strategies.

Thyroid Fine-Needle Aspiration an AI

Torres et al [41] recalls that yhyroid cytology is a rapidly evolving field, marked by significant advances aimed at accurately diagnosing thyroid nodules, distinguishing between benign and malignant lesions, and stratifying risk when a definitive diagnosis is challenging. Fine-needle aspiration (FNA) is a key method for diagnosing thyroid nodules, supported by standardized reporting systems such as the Bethesda System for Reporting Thyroid Cytopathology, which classifies nodules into various risk categories.Recent advancements include the integration of molecular testing, which stratifies patients into low, intermediate, or high-risk categories based on malignancy probability, aiding in treatment decisions. Additionally, artificial intelligence (AI) is increasingly being incorporated into thyroid cytology to further enhance diagnostic accuracy and efficiency. AI technologies, such as machine learning algorithms and image analysis tools, are used to analyze cytological images and molecular data. These AI systems can identify patterns and features that might be missed by human observers, provide more precise risk assessments, and support decision-making processes by integrating large volumes of data. By combining AI with traditional and molecular diagnostic methods, thyroid cytology is poised to achieve even greater accuracy and reliability in diagnosing and managing thyroid nodules.

S.2 Analytical summaries for *primary studies* [42-55] in section 4

In the following the analytical summarie for the primary cutting-edge studies selecet.

[42] Liu TJ, et al. (2024)

This study evaluated the AIxURO platform's effectiveness in enhancing urine cytology for bladder cancer diagnosis. AIxURO was compared with traditional methods (microscopy and WSI review) using 116 urine cytology slides. The AI tool improved diagnostic accuracy significantly, with sensitivity for atypical urothelial cells rising from 25.0%-30.6% to 63.9%, and for suspicious high-grade urothelial carcinoma from 15.2%-27.3% to 33.3%. AIxURO also improved positive and negative predictive values and reduced screening times by 52.3%-83.2% compared to microscopy and 43.6%-86.7% compared to WSI review. The study concludes that AIxURO has substantial potential to enhance both the accuracy and efficiency of bladder cancer diagnostics.

[43] Zhao D, et al. (2024)

This study assessed the performance of the ResNeSt deep convolutional neural network in distinguishing papillary thyroid carcinomas from benign nodules in cases with atypia of undetermined significance (AUS). The ResNeSt model achieved an accuracy of 92.49% on fragmented images and 84.78% on a patient-wise basis. The model demonstrated high sensitivity (95.79%) and specificity (88.46%). It was particularly effective in analyzing cell nuclei characteristics, showing that malignant nodules had larger and deeper stained nuclei compared to benign nodules. The study indicates that ResNeSt has strong potential for improving the diagnostic accuracy of fine-needle aspiration biopsy in thyroid nodules.

[44] Mhaske S, et al. (2024)

This research focused on the use of machine learning algorithms to automate the analysis of nuclear parameters in oral exfoliative cytology. The study compared the performance of convolutional neural networks (CNN) and support vector machines (SVM) in analyzing cytological images from 60 patients, split between those with and without clinically suspicious oral malignancy. Significant differences in nuclear size, shape, and chromatin distribution were found between the study and control groups. SVM demonstrated a Pearson correlation coefficient of 0.6472, while CNN showed a higher correlation of 0.7790. The study suggests that AI models like SVM and CNN could improve early detection rates and overall diagnostic accuracy in oral cytology.

[45] Kim T, et al. (2023)

This study evaluated a deep learning model for diagnosing lung cancer using a nationwide dataset of respiratory cytology images. The Densenet121 model, among six convolutional neural networks tested, provided the highest performance, achieving sensitivity of 95.9%, specificity of 98.2%, and accuracy of 96.9%. The model significantly improved pathologists' diagnostic accuracy from 82.9% to 95.9% and enhanced inter-observer agreement. This research highlights the potential of deep learning to boost diagnostic accuracy and consistency in lung cancer diagnosis from respiratory cytology.

[46] Park HS, et al. (2023)

The study explored the use of deep learning for diagnosing metastatic breast carcinoma in pleural fluid cytology. A deep convolutional neural network (Inception-ResNet-V2) was trained on a large dataset of 569 cytological slides and 34,221 image patches. The model outperformed pathologists in accuracy (81.1% vs. 68.7%), sensitivity (95.0% vs. 72.5%), and specificity (98.6% vs. 88.9%). After reviewing discordant cases, pathologists' accuracy improved, but the deep learning model still demonstrated superior performance. The study indicates that AI can significantly enhance diagnostic accuracy in metastatic breast cancer cytology.

[47] Ozer E, et al. (2023)

This study investigated the use of deep learning for intraoperative cytological diagnosis of brain tumors. A deep neural network was trained to classify biopsy material from 205 cases, including various brain lesions. The model achieved high diagnostic accuracy of 95% for patch-level and 97% for patient-level classification. The study suggests that deep learning models could be a valuable tool for providing rapid and accurate intraoperative diagnoses of CNS tumors, complementing traditional methods.

[48] Saikia AR, et al. (2019)

The paper compared various convolutional neural network (CNN) architectures for classifying breast fine needle aspiration cytology (FNAC) images. Using a dataset of 212 images (augmented to 2120), the study evaluated models including VGG16, VGG19, ResNet-50, and GoogLeNet-V3. GoogLeNet-V3 achieved the highest accuracy of 96.25%. This comparison demonstrates the potential of CNN models to improve diagnostic accuracy and consistency in breast cancer FNAC.

[49] Sanyal P, et al. (2018)

This study developed a neural network to identify papillary thyroid carcinoma from fine-needle aspiration cytology (FNAC) smears. The neural network was trained on 186 images of papillary carcinoma and 184 images of other thyroid lesions. It achieved good sensitivity (90.48%), moderate specificity (83.33%), and a high negative predictive value (96.49%). The study highlights the potential of neural networks to automate and enhance the accuracy of thyroid FNAC diagnoses, though further training is needed to address limitations.

[50] Peñaranda F, et al. (2018)

This study investigated the use of Fourier transform infrared (FTIR) spectroscopy to differentiate between various cultured skin cell lines, including non-tumoral and tumoral types. A total of 22,700 single skin cells were analyzed across four cell lines, with cells prepared in three distinct batches. The research explored different spectral preprocessing and classification techniques to address Mie scattering artifacts and optimize learning models. The algorithms achieved high balanced accuracy (0.85 to 0.95) when trained and tested within the same batch. However, when trained and tested with cells from different batches, the balanced accuracy dropped to 0.35 to 0.60, highlighting the impact of sample preparation on classification performance. The study emphasizes the need for careful handling of sample preparation to ensure reliable FTIR spectroscopy results.

[51] Kumar N, et al. (2020)

This review discusses the current perspectives and future directions of Whole Slide Imaging (WSI) in pathology. WSI has been validated for numerous applications and recently received FDA approval for primary surgical pathology diagnosis, marking a significant milestone. The review covers advancements in digital scanners, image visualization, and AI integration, which enhance WSI's utility. Despite benefits such as improved accessibility and reduced physical storage requirements, challenges including high costs, technical issues, and resistance to new technologies persist. The article provides a comprehensive overview of WSI technology, its applications, limitations, and future potential, serving as a guide for students and pathologists.

[52] Liu W, et al. (2022)

This study evaluated the effect of cell aspect ratio on deep learning models used for classifying cervical cytopathology images. The research compared the performance of deep learning methods on datasets with standardized and scaled images. Results indicated that deep learning models are robust to changes in cell aspect ratio, with consistent classification performance across varying dimensions. The study highlights that despite the importance of aspect ratios in clinical diagnostics, deep learning models can effectively manage and classify images even with aspect ratio variations, validated using both the SIPaKMeD and Herlev datasets.

[53] Chen P, et al. (2021)

The study introduced an automatic diagnosis framework for whole slide pathology images using unit stochastic selection and attention fusion techniques. The framework involves training a unit-level convolutional neural network (CNN) to extract features and estimate non-benign probabilities of units, followed by stochastic selection and attention fusion to form a fixed-length descriptor for slide diagnosis. Evaluations on thyroid, colonoscopy, and cervical pap smear slides demonstrated high accuracy (greater than 0.8) and AUC values (greater than 0.85) for the framework, indicating its effectiveness and potential for clinical application in diagnosing various types of pathology slides.

[54] Zhou T, et al. (2024)

This study compared the diagnostic performance of an AI-assisted system for thyroid nodules with traditional fine needle aspiration (FNA) cytopathology and BRAFV600E mutation analysis. The AI system was evaluated using ultrasound images from 637 nodules and compared against FNA and mutation analysis in terms of sensitivity, specificity, and accuracy. Results showed that the AI system performed similarly to FNA combined with mutation analysis in accuracy. The study suggests that AI offers advantages in operability, time efficiency, and non-invasiveness, making it a viable alternative for thyroid nodule diagnosis and potentially reducing the need for invasive FNA biopsies.

[55] Sohn A, et al. (2023)

This study proposed a deep learning framework, MIPCL, for triaging and predicting adenocarcinoma in pancreatic cytology whole slide imaging. The framework demonstrated superior performance compared to existing models, with an F1-Score of 91.07% and AUROC of 0.9435. The study also presented a dataset curation strategy that increased the number of training examples. MIPCL not only improved predictive accuracy but also highlighted key regions on slides contributing to the final diagnosis, showing its potential as an effective screening tool in pancreatic cancer diagnosis.
